# Oncogenic EML4-ALK assemblies suppress growth factor perception and modulate drug tolerance

**DOI:** 10.1038/s41467-024-53451-7

**Published:** 2024-11-02

**Authors:** David Gonzalez-Martinez, Lee Roth, Thomas R. Mumford, Juan Guan, Anh Le, Robert C. Doebele, Bo Huang, Asmin Tulpule, Magdalena Niewiadomska-Bugaj, Trever G. Bivona, Lukasz J. Bugaj

**Affiliations:** 1 Department of Bioengineering, University of Pennsylvania, Philadelphia, PA 19104 USA; 2 Department of Physics, Department of Anatomy and Cell Biology, University of Florida, Gainesville, FL 32611 USA; 3 Department of Anatomy and Cell Biology, University of Florida, Gainesville, FL 32611 USA; 4 Division of Medical Oncology, University of Colorado School of Medicine, Aurora, CO 80045 USA; 5 Department of Pharmaceutical Chemistry, UCSF, San Francisco, 94143 USA; 6 Department of Biochemistry and Biophysics, UCSF, San Francisco, 94143 USA; 7 Chan Zuckerberg Biohub, San Francisco, 94158 USA; 8 Department of Pediatrics, Memorial Sloan Kettering Cancer Center, New York, NY 10065 USA; 9 Department of Statistics, Western Michigan University, Kalamazoo, MI 49008 USA; 10 Department of Medicine, Division of Hematology and Oncology, UCSF, San Francisco, CA 94143 USA; 11 Abramson Cancer Center, University of Pennsylvania, Philadelphia, PA 19104 USA; 12 Institute of Regenerative Medicine, University of Pennsylvania, Philadelphia, PA 19104 USA

**Keywords:** Cancer therapeutic resistance, Growth factor signalling

## Abstract

Drug resistance remains a challenge for targeted therapy of cancers driven by EML4-ALK and related fusion oncogenes. EML4-ALK forms cytoplasmic protein condensates, which result from networks of interactions between oncogene and adapter protein multimers. While these assemblies are associated with oncogenic signaling, their role in drug response is unclear. Here, we use optogenetics and live-cell imaging to find that EML4-ALK assemblies suppress transmembrane receptor tyrosine kinase (RTK) signaling by sequestering RTK adapter proteins including GRB2 and SOS1. Furthermore, ALK inhibition, while suppressing oncogenic signaling, simultaneously releases the sequestered adapters and thereby resensitizes RTK signaling. Resensitized RTKs promote rapid and pulsatile ERK reactivation that originates from paracrine ligands shed by dying cells. Reactivated ERK signaling promotes cell survival, which can be counteracted by combination therapies that block paracrine signaling. Our results identify a regulatory role for RTK fusion assemblies and uncover a mechanism of tolerance to targeted therapies.

## Abstract (web summary)

The EML4-ALK oncogene forms  cytoplasmic protein condensates that are associated with its oncogenic signaling. Here, the authors demonstrate that these EML4-ALK assemblies also sequester receptor tyrosine kinase (RTK) adapter proteins suppressing signaling which was reversed upon ALK inhibition, resulting in rapid resensitisation to growth factors and tumor cell survival.

## Introduction

Drug resistance remains a central challenge that prevents durable cancer treatment, including for the large cohort of cancers driven by RTK fusion oncogenes^1^. EML4-ALK (echinoderm microtubule-associated protein-like 4-anaplastic lymphoma kinase) is a common RTK fusion that drives ~5–7% of non-small cell lung cancer (NSCLC)^2–4^. EML-ALK+ cancers exhibit oncogene addiction, whereby inhibition of ALK signaling causes cell death and tumor shrinkage^5^. Nevertheless, resistance and tumor relapse inevitably emerge^6,7^, highlighting the urgent need to better understand the functional interactions between oncogenes, host cells, and drugs in order to achieve more effective and durable therapeutic response.

**Fig. 1:**
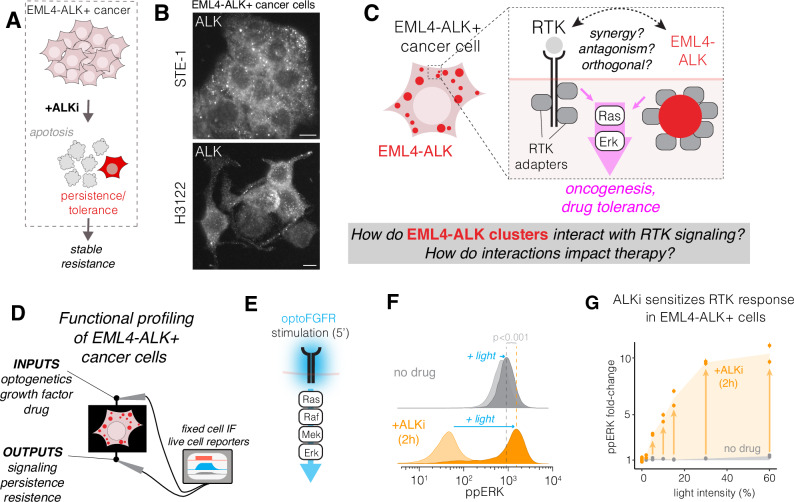
Optogenetic probing of EML4-ALK+ cancer cells reveals suppression of RTK signaling. **A** EML4-ALK+ cancer cells treated with ALK inhibitors (ALKi) can persist through therapy and acquire stable drug resistance. **B** Immunofluorescence shows punctate expression of ALK in two EML4-ALK+ cancer cell lines. Scale = 10 µm. **C** Understanding functional interactions between EML4-ALK and transmembrane RTKs. **D** Functional profiling of RTK signaling in EML4-ALK+ cancer cells. **E** OptoFGFR allows blue-light-induced stimulation RTK/ERK signaling. **F** Single-cell immunofluorescence of ppERK levels in STE-1 cells stimulated with light (optoFGFR) in the presence (orange) or absence (gray) of ALK inhibitor crizotinib (ALKi, 1 µM). Significance assessed using one-sided KS test for difference of two distributions. **G** ppERK fold-change in response to 5 min of blue light stimulation over the range of the indicated stimulus intensities. Data points show the ratio of ppERK from stimulated and unstimulated cells. Each data point in (**G**) shows the mean of 3000–5000 cells. Error bars = 95% CI. See Supplementary Table 1 for biological and experimental replicate numbers for all experiments.

EML4-ALK drives cancer primarily through sustained RAS/ERK signaling^8^. Although RAS/ERK-driven cancers respond to therapeutic blockade of the pathway, treatment is challenged by robust autoinhibitory feedback loops that reactivate the pathway after its inhibition. In certain melanomas and colorectal cancers, inhibition of mutant BRAF suppresses oncogenic ERK signals but simultaneously relieves ERK-dependent negative feedback of RTKs, resulting in strong epidermal growth factor receptor (EGFR) stimulation, cell survival, and drug resistance^9–12^. For EML4-ALK+ cancers, reactivation of RTKs after ALK inhibition similarly promotes drug tolerance and acquired resistance^8,13–18^ (Fig. 1A), although the mechanisms of reactivation are not well understood.

Multiple variants of EML4-ALK form large cytoplasmic protein condensates in cancer cells^8,19,20^ (Fig. 1B). Here, the term “condensate” is a general descriptor of higher-order protein assemblies that form through networks of multivalent interactions^21–23^. Such multivalent assemblies can exhibit a range of sizes, biophysical properties, and routes of formation. For EML4-ALK, condensation is linked to its signaling: multimerization of the EML4 fragment leads to activation and tyrosine phosphorylation of the ALK fragment. This phosphorylation recruits RTK adapters like GRB2 that can also multimerize. The combined multivalency of the oncogene and adapters yields interaction networks that can result in large, micron-scale condensates^19^.

While multivalent EML4-ALK/adapter assemblies are required for oncogenic signaling^19^, it is unclear whether they may have additional functional impacts on signal transduction and drug response. Of particular interest are interactions with transmembrane RTKs including EGFR (Fig. 1C), which is expressed in over 90% of NSCLC and is correlated with poor prognosis^24^ and drug resistance^9,13,15,25–28^.

We recently developed an approach called ‘optogenetic functional profiling’ to detect how oncogene expression can corrupt cell signal transmission^29,30^. In this black-box approach, we apply precise signaling inputs, for example, using light-activated optogenetic probes, and we quantify differential downstream outputs (e.g., signaling, transcription, cell fate) as a function of oncogene expression or drug treatment. Previously, this approach revealed that certain BRAF-mutant cancer cells and drug-treated cells exhibited abnormally slow RAS/ERK activation kinetics that could cause cells to misinterpret dynamic stimuli leading to hyperproliferation^29^.

In the present work, we apply optogenetic profiling to uncover functional interactions between EML4-ALK assemblies and transmembrane RTK signaling (Fig. 1D). We first discover that EML4-ALK expression dramatically suppresses a cell’s perception of RTK signals. Suppression results not from biochemical feedback, but rather from sequestration of RTK adapters within assemblies of active EML4-ALK. Suppression is relieved within minutes of ALK inhibition, resulting in rapid resensitization of RTKs to external growth factors. In cancer cells, drug-induced resensitization enables perception of paracrine ligands from neighboring cells that reactivate EGFR/ERK within ~1 h after drug treatment. These paracrine signals suppress cell killing and permit tolerance to ALK inhibitors. Our work thus uncovers an important role for EML4-ALK condensates in signal regulation and drug response and suggests candidate co-targets for more durable ALK inhibitor therapy.

## Results

### Optogenetic profiling reveals that RTK signaling is suppressed in EML4-ALK+ cancer cells

To determine whether EML4-ALK could alter RTK signal transmission, we expressed a light-sensitive fibroblast growth factor receptor (optoFGFR^31^) in STE-1 cancer cells, which express EML4-ALK variant 1 (V1)^32^ (Fig. 1E, Supplementary Fig. S1A). We observed signal transmission by applying blue light stimuli and measuring pathway activation (phospho-ERK, ppERK) in the presence or absence of ALK activity. In these cells, light stimulation of optoFGFR induced only minimal ppERK signal increase above the high basal levels driven by EML4-ALK (Fig. 1F, top). Surprisingly, pretreatment with ALK inhibitor crizotinib (ALKi, 1 µM) led to exceedingly strong ERK signaling, consistently surpassing levels achieved in the absence of drug treatment (Fig. 1F, bottom). The dynamic range (fold-change) of signal induction increased as a function of light intensity and reached a maximum of ~10-fold at the highest levels of light stimulation, compared to ~1.5-fold in the absence of drug (Fig. 1G). Notably, strong ppERK increase in drug-treated cells was observed in response to even low levels of light (8 mW/cm^2^), a level that did not provide measurable increase in untreated cells, suggesting that ALK inhibition can sensitize cancer cells to weak RTK signals (Supplementary Fig. S1B). We note that although the magnitude of dynamic range increase was dependent on the expression levels of optoFGFR, ALKi potentiated signaling across all expression levels (Supplementary Fig. S1C). Collectively, these results indicate a strong suppressive effect of EML4-ALK activity on transmembrane RTK signaling.

### EML4-ALK suppresses—and ALK inhibition restores—EGFR signaling

**Fig. 2:**
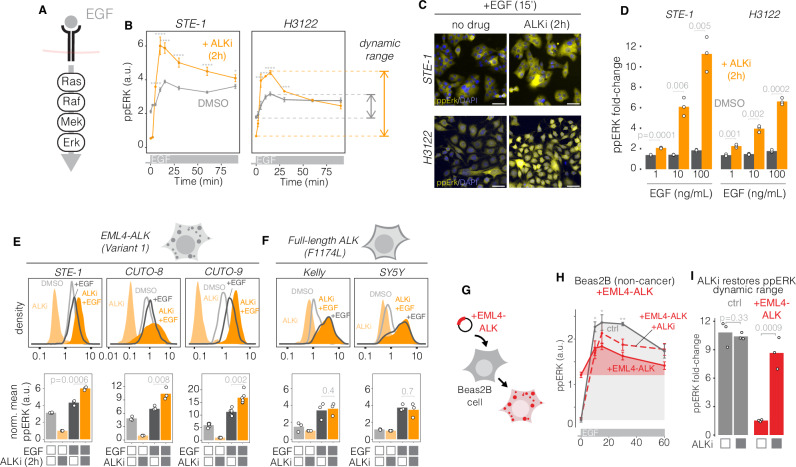
EML4-ALK suppresses EGFR signaling. **A** EGF stimulates EGFR and downstream RAS/ERK signaling. **B** ppERK levels in response to EGF (100 ng/mL) in the presence of 1 µM ALKi (orange) or DMSO (gray) in STE-1 and H3122 cancer cells. Data points represent mean of 1000–2800 cells for STE-1 and 800–3000 cells for H3122. Error bars = 95% CI. ****p < 0.0001, ***p = 0.0007, *p = 0.02 by one-sided T-test comparing ALKi vs DMSO. *n* = 3 biological replicates. **C** Representative images of ALKi-dependent potentiation of ERK response to EGF (**B**). **D** Fold-change increase over a range of EGF concentrations. Data represent ratio of mean ppERK from EGF-stimulated (15 min) vs unstimulated cells. Significance assessed using one-sided T-test, *n* = 3 biological replicates. **E**, **F** Representative single-cell (top) and average (bottom) ERK responses to EGF (50 ng/mL, 15 min) and ALKi (crizotinib, 1 μm, 2 h pretreatment) in two primary patient-derived cell lines, CUTO-8 and CUTO-9, that harbor EML4-ALK(V1) (**E**), or in cell lines driven by a constitutively active, full-length ALK mutant (**F**). Data points in (**E**, **F**) (bottom) represent mean ppERK normalized to mean ppERK of ALKi-treated cells for 900–2100 STE-1, 900–1500 CUTO-8, 200–300 CUTO-9, 200–600 Kelly cells and 1700–2700 SY5Y cells. Significance assessed by one-sided T-test, *n* = 3 biological replicates for STE-1, CUTO-8, Kelly, and SY5Y; *n* = 6 biological replicates for CUTO-9. **G** EML4-ALK(V1) (EML4-ALK-2A-H2B-miRFP) or a control construct (H2B-miRFP) were transiently expressed in lung epithelial Beas2B cells. **H** Time course of ppERK immunofluorescence levels in response to EGF stimulation (50 ng/mL). Data points represent mean ± SEM of 120–300 cells for untransfected Beas2B and 80–160 cells for Beas2B expressing EML4-ALK. **p = 0.003, *p = 0.04, by one-sided T-test for ppERK in transfected vs untransfected cells, *n* = 3 biological replicates. **I** Dynamic range of ppERK in EML4-ALK-expressing Beas2B in response to EGF in the presence or absence of ALKi (1 µM) pretreatment. Significance assessed using one-sided T-test, *n* = 3 biological replicates.

EGFR is found in >90% of NSCLC and modulates drug responses and resistance development in EML4-ALK+ cancers^10,12–14,24^. To determine whether EML4-ALK could generally suppress endogenous transmembrane RTKs including EGFR, we measured ppERK induction upon addition of epidermal growth factor (EGF) in STE-1 and H3122 cells, two established EML4-ALK (V1)+ cell lines^32^ (Fig. 2A). Strong (100 ng/mL) EGF stimulation gave minimal ppERK response in the absence of drug, but pretreatment with ALKi substantially increased both the absolute magnitude as well as fold-change of ppERK in both cell lines (Fig. 2B–D, Supplementary Fig. S2A, B). As before, increased dynamic range was due to lower baseline—but also higher maximal—ppERK signaling and the magnitude of fold-change increase was dose-dependent (Fig. 2D). We found similar responses using first-line ALK inhibitor alectinib (Supplementary Fig. S2C) as well as in two additional primary patient-derived cell lines that express EML4-ALK (V1) (Fig. 2E, Supplementary Fig. S2D, E). However, EGF response was not potentiated in drug-treated cancer lines that expressed constitutively active transmembrane ALK mutants (Fig. 2F). Thus, suppression of EGFR signaling is a shared property of EML4-ALK(V1)+ cancer cells and depends on oncogene activity, but is not a general property of oncogenic ALK receptor activity.

To test the sufficiency of EML4-ALK for RTK suppression, we measured EGF response in isogenic, non-transformed lung epithelial Beas2B cells that were transfected with EML4-ALK (V1) (Fig. 2G). Exogenous EML4-ALK expression raised basal ppERK levels (t = 0, Fig. 2H), but EGF stimulation resulted in only a small increase in ppERK relative to cells transfected with a control plasmid, indicating suppression of EGFR/ERK signaling. Pre-incubation with ALKi reversed suppression and allowed stronger maximum and fold-change of ERK response (Fig. 2H, I). Collectively, our results show that EML4-ALK(V1) activity is sufficient to desensitize cellular response to EGFR stimuli and that ALK inhibition resensitizes and potentiates this response.

#### Mapping EGFR suppression using optogenetics

**Fig. 3:**
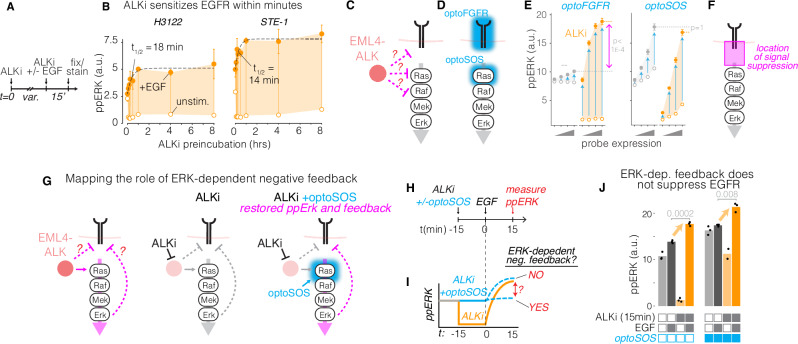
Mapping RTK feedback suppression using optogenetics. **A** Time course of EGFR sensitization was obtained by pre-incubating cancer cells with 1 µM crizotinib for a variable period before stimulation with EGF (50 ng/mL) for 15 min, followed by fixation and immunostaining for ppERK. **B** ppERK induction as a function of ALKi pre-incubation time. Open circles: unstimulated; closed circles: EGF-stimulated. Data points represent mean of three biological replicates, each representing 1200–2700 cells for STE-1 and 2000–3300 cells for H3122. Error bars = 95% CI. **C** Pinpointing the location of EML4-ALK interaction with RTK/ERK signaling. **D** OptoFGFR and optoSOS permit optogenetic stimulation at successive nodes of the pathway. **E** Quantification of ppERK levels in response to optoFGFR or optoSOS in the presence (orange) or absence (gray) of ALKi (1 µM crizotinib). *X*-axis represents optoFGFR or optoSOS expression quartiles. Data points represent mean ± SEM of three biological replicates, each representing 200–1100 cells. Significance assessed using one-sided T-test, *n* = 3 biological replicates. Open circles: unstimulated; closed circles: light stimulated. **F** ALK-dependent suppression is observed only with optoFGFR, suggesting that suppression happens upstream of RAS but downstream of RTK activation. **G** Testing the role of ERK-dependent negative feedback on RTK suppression. Light stimulation of optoSOS drives elevated levels of ppERK during ALKi treatment and sustains any potential ERK-dependent negative feedback that would otherwise be lost during ALK inhibition. **H** STE-1 cells were treated with either ALKi or ALKi and optoSOS stimulation, and the response to EGF was assessed. **I** Predicted results and implications for ERK-dependent feedback. **J** optoSOS did not suppress ALKi-induced potentiation of EGF response, suggesting that ERK-dependent negative feedback does not account for EGFR suppression. Data points represent means of 150–900 cells per condition. Significance assessed using one-sided T-test, *n* = 3 biological replicates.

We next sought to understand the molecular mechanism by which EML4-ALK suppressed EGFR signaling. We confirmed that ALKi suppressed RAS/ERK signaling but did not alter levels of EML4-ALK (Supplementary Fig. S3A). To narrow candidate mechanisms of suppression, we first determined the duration of ALKi pre-incubation that was required to observe enhanced EGFR/ERK signaling. We preincubated both STE-1 and H3122 cells with 0–8 h of ALKi, and we analyzed ppERK levels in response to subsequent EGF stimulation (Fig. 3A). In both cell lines, an increase in ppERK amplitude was observed with as little as 5 min of ALKi pre-incubation (20 min total including stimulation) and rose to half-max with only ~15 min of pre-incubation (Fig. 3B). Such fast response suggested a primarily post-translational mechanism.

To pinpoint the molecular interactions that mediated EGFR suppression, we measured ppERK response after targeted optogenetic stimulation at successive nodes of the EGFR/ERK pathway^30^ (Fig. 3C, D). We stimulated STE-1 cell lines that stably expressed either optoFGFR or optoSOS, which allows activation of RAS/ERK signals through light-induced membrane recruitment of the SOS catalytic domain^33,34^ (Fig. 3C, D, Supplementary Fig. S3B, C). As before, optoFGFR was strongly suppressed across all expression levels of the probe, and ALKi pretreatment potentiated signaling (Fig. 3E, left). By contrast, optoSOS-driven signaling was not suppressed at any expression level, and ALKi pretreatment had no effect on the maximal signal achievable (Fig. 3E, right). These results suggest that EML4-ALK suppresses signaling upstream of RAS stimulation, for example, at the receptor level. However, ALKi pre-incubation did not potentiate phosphorylation of EGFR upon EGF stimulation (Supplementary Fig. S3D, E). Collectively, our results show that EML4-ALK suppresses RTK signaling downstream of receptor phosphorylation but upstream of RAS activation. These results implicate a role for the adapter proteins that couple these two successive nodes (Fig. 3F).

Rapid post-translational negative feedback of RTKs has been described previously, for example through ERK-dependent phosphorylation of SOS1^11,35^. To test the role of ERK-dependent negative feedback in our system, we decoupled ALK inhibition from the loss of ERK signaling by supplementing cells with optogenetic RAS (optoSOS) stimulation during ALK inhibition (Fig. 3G). We assessed response to EGF after 15 min of ALKi pre-incubation, a duration that was sufficiently long to resensitize EGFR (Fig. 3B) but sufficiently short to test only post-translational effects of ERK inhibition. One experimental group received oncogenic levels of optogenetic RAS/ERK signaling during the 15 min of ALKi treatment in order to maintain any ERK-dependent feedback that would have been otherwise lost through ALK inhibition (Fig. 3G–I, Supplementary Fig. S4A). Optogenetic signal supplementation did not diminish the enhanced response to EGF (Fig. 3J). Similar results were obtained after 1 and 2 h of ALKi pre-incubation (Supplementary Fig. S4B). In agreement, the levels of ERK-dependent negative regulator Spry2 did not change after acute ALKi pre-incubation (Supplementary Fig. S4C). These results show that, in EML4-ALK+ cancer cells, EGFR suppression and rapid drug-induced resensitization are not mediated by established, ERK-dependent mechanisms.

### Active EML4-ALK assemblies suppress EGFR through sequestration of RTK effectors

**Fig. 4:**
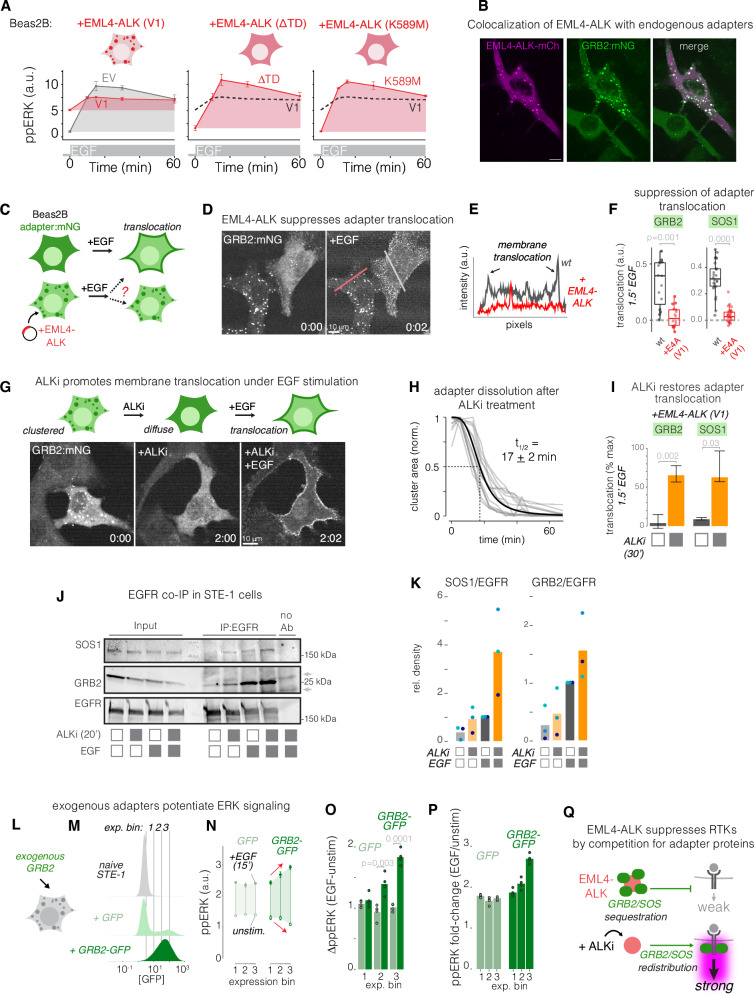
EML4-ALK assemblies suppress EGFR through sequestration of RTK effectors. **A** Quantification of ppERK response to EGF (50 ng/mL) in Beas2B cells that were transfected with EML4-ALK (V1), EML4-ALK (∆TD), or kinase-dead EML4-ALK (K589M), or with an empty vector (EV). Data points represent mean ± SEM of three biological replicates, each representing 200–400 EV cells, 130-270-EML4-ALK (V1) cells, 55–160 EML4-ALK (∆TD) cells, or 260–450 EML4-ALK (K589M) cells. **B** Colocalization of EML4-ALK condensates with endogenously tagged GRB2 (*GRB2:mNG2*). See Fig. 5 for quantitation. Scale = 10 µm. **C** *GRB2:mNG2* Beas2B cells were transiently transfected with EML4-ALK and stimulated with EGF (50 ng/mL) to visualize GRB2 translocation in the presence and absence of EML4-ALK (V1). **D** Impaired membrane translocation of GRB2 in the presence of EML4-ALK condensates. Time in mm:sec. See Supplementary Movie 1. **E** Line scan of GRB2 intensity distribution in the presence (red) or absence (gray) of EML4-ALK expression, as depicted in (**C**). **F** Quantitation of translocation of endogenous GRB2 or SOS1 in the presence (red) or absence (gray) of EML4-ALK. Boxplot indicates the median and upper/lower quartiles, and whiskers extend to 1.5*IQR. See Fig. 5 for full quantitation. **G** GRB2 localization and translocation were visualized upon treatment with 1 µM ALKi and subsequent stimulation with EGF (50 ng/mL). Time in hh:mm. **H** Quantification of kinetics of GRB2 dissociation from condensates after ALKi treatment. **I** ALKi restores GRB2 and SOS translocation. Plot shows median translocation of endogenous adapters in cells expressing EML4-ALK(V1) represented as a fraction of translocation in the absence of EML4-ALK (V1). Data represent medians, error bars show 1st and 3rd quartiles of 1000 bootstrapped samples (distributions found in Fig. 5F, G). Significance assessed by one-sided bootstrap test for comparison of medians. See Fig. 5F, G for underlying data and quantitation. **J** Immunoprecipitation of EGFR shows enhanced co-precipitation of GRB2 and SOS1 in the presence of both ALKi pretreatment and EGF in STE-1 cells. gray arrows: non-specific bands. **K** Densitometry quantification of three independent pulldowns. **L** Testing effect of GRB2 overexpression on ERK response. **M** Expression levels of GRB2-GFP or GFP analyzed in (**N**, **O**). **N** ppERK levels in the absence (open circles) or presence (closed circles) of EGF stimulation (50 ng/mL) as a function of expression levels of GFP or GRB2-GFP. Data represent mean ± SEM of three biological replicates, each representing the mean of 100–300 cells. **O** Absolute magnitude of ppERK increase for each expression bin from data shown in (**N**). Significance assessed by one-sided T-test, *n* = 3 biological replicates. **P** Fold-change of response calculated from data in (**N**). **Q** Conceptual model of how EML4-ALK suppresses transmembrane RTKs. EML4-ALK sequesters adapters like GRB2/SOS1 and prohibits their translocation to activated RTKs. ALK inhibition releases adapter sequestration and restores cellular response to RTK stimulation.

Protein condensates can act as a negative regulator in both natural and engineered systems when they sequester essential components of biochemical reactions^36–38^. To test if EML4-ALK assemblies suppressed RTKs, we first examined EGF response in cells transfected with EML4-ALK mutants that failed to assemble condensates due to mutations in either the trimerization domain (ΔTD) or the kinase domain (K589M)^19^. In contrast to wt EML4-ALK (V1) (Fig. 4A, left), the condensate-deficient mutants did not stimulate ERK signaling and permitted higher levels of EGF-induced ERK activity (Fig. 4A, middle and right), suggesting that signaling-competent EML4-ALK assemblies were essential for suppression of EGFR sensitivity.

We hypothesized that EGFR suppression could result from cytoplasmic sequestration of RTK adapter proteins. Such adapters, including GRB2 and SOS1, are required to transmit signals from both EML4-ALK and EGFR^19^ and thus represent shared resources that could implement competitive inhibition. We first directly observed the association of adapters with EML4-ALK using Beas2B cells where GRB2 was fluorescently tagged at the endogenous locus^19^. Upon transfection with mCh-EML4-ALK, GRB2 colocalized within EML4-ALK condensates (Fig. 4B), as shown previously^19,20^. We then measured translocation of GRB2 in response to EGF stimulation (Fig. 4C). In untransfected cells, GRB2 appeared diffuse in the cytoplasm but translocated to the membrane within <1 min of EGF addition (Fig. 4D–F, Supplementary Movie 1). However, in cells with EML4-ALK, GRB2 remained sequestered in the cytoplasmic puncta and did not translocate in response to EGF (Fig. 4D–F Supplementary Movie 1). Such sequestration was also observed for SOS1 (Fig. 4F). Upon treatment of EML4-ALK-expressing cells with ALKi, adapters rapidly diffused from condensates into the cytoplasm (t_1/2_ = 17 ± 2 min) (Fig. 4G, H, Supplementary Movie 2). Subsequent treatment with EGF now stimulated robust membrane translocation of both GRB2 and SOS1 despite the presence of exogenous EML4-ALK (Fig. 4G, I). Of note, ALKi treatment does not cause complete dissolution of the underlying granule-like EML4-ALK condensates in cancer cells, though their number and size diminish, consistent with previous results^20^ (Supplementary Fig. S5A, B).

We next measured the ability of EGFR to recruit adapters in EML4-ALK+ cancer cells through co-immunoprecipitation with endogenous EGFR (Fig. 4J, K, Supplementary Figs. S5C, D, S6). In both STE-1 and H3122 cells, a short (20 min) ALKi pre-incubation yielded dramatically stronger co-precipitation of both GRB2 and SOS1 with EGFR in response to EGF stimulation compared to cells that received only ALKi or only EGF (Fig. 4J, K, Supplementary Figs. S5C, D, S6).

If adapters are sequestered in EML4-ALK assemblies, then supplementing the cell with exogenous adapters could restore sensitivity to EGFR signaling (Fig. 4L). Overexpression of GRB2-GFP exerted effects on both basal and stimulated EML4-ALK cancer cells, in a manner dependent on GRB2 expression levels (Fig. 4L–P). In STE-1 cells, exogenous GRB2-GFP both lowered basal ppERK levels and potentiated maximal ppERK response to EGF relative to GFP controls, resulting in an increase in absolute magnitude as well as fold-change of response (Fig. 4O, P). We speculate that decreased basal and modest increase in stimulated ppERK occurs because the exogenous GRB2 contributes not only to the diffuse cytoplasmic compartment but also interacts with active assemblies, altering their stoichiometry. Similar effects were observed in H3122 cells, though with smaller relative increase in absolute and fold-change ppErk response relative to GFP controls (Supplementary Fig. S5E–H). We hypothesized that, in these cells, the more modest increase could be due to continued limitation of another component, e.g., SOS1, which is expressed at ~10–100-fold lower concentration than endogenous GRB2^39,40^. In agreement, transient expression of SOS1 further potentiated ppErk response in these cells (Supplementary Fig. S5I, J).

Collectively, our results show that active, multivalent EML4-ALK assemblies sequester adapters including GRB2 and SOS1 and suppress their translocation to the membrane upon EGF stimulation, providing a mechanism by which EML4-ALK can competitively suppress EGFR signaling. ALKi treatment restores the available pool of adapters and consequently restores EGFR transmission (Fig. 4Q). Because this model relies on general properties of RTK fusions (e.g., multivalency and adapter recruitment), it predicts that cancer cells driven by other RTK fusions that form condensates would similarly show repressed EGFR signaling. We thus measured EGF response in TPC-1 cells, which harbor the CCDC6-RET fusion. Like EML4-ALK, CCDC6-RET can form condensates that colocalize with many RTK adapters, including GRB2 and SOS1^19^. As predicted, pretreatment with RET inhibitor BLU-667 permitted stronger response to EGF compared to that observed in drug-naive cells (Supplementary Fig. S7A, B). Furthermore, we tested a panel of cancer cell lines driven by either hyperactivating mutants or amplification in EGFR or HER2 and found no similar hyperactivation of transmembrane RTK signaling upon oncogene inhibition, further linking signal suppression specifically to cytoplasmic RTK assemblies and not simply to hyperactivated RTKs (Supplementary Fig. S7C, D).

### Adapter sequestration and signal suppression is a common property of EML4-ALK variants

**Fig. 5:**
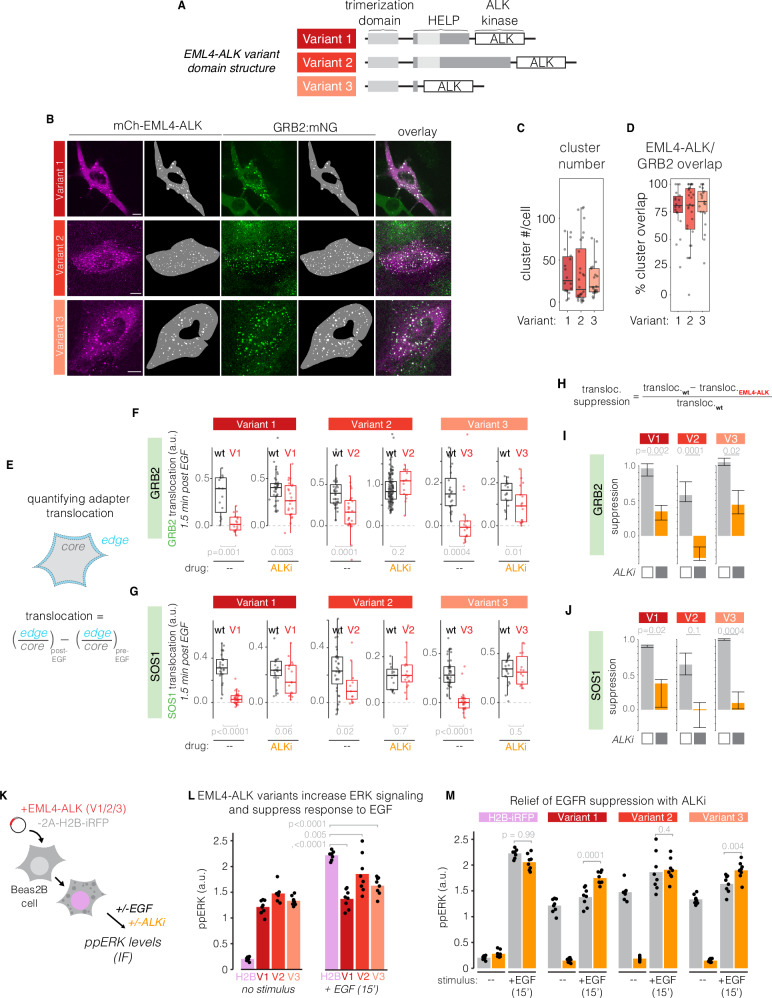
Condensation and suppression of RTK signaling are common properties among EML4-ALK variants. **A** Three common oncogenic variants of EML4-ALK (Variants 1–3) share a common ALK fragment but differ in the lengths of the EML4 domain. **B** Expression of mCh-EML4-ALK(V1/2/3) in *GRB2:mNG2* Beas2B cells showed condensation of each variant as well as the propensity of the condensates to colocalize with GRB2. Scale = 10 µm. **C** Quantification of puncta per cell for each variant. **D** Quantification of the percent of EML4-ALK puncta that overlap with GRB2 puncta in each cell. Boxplots in (**C**, **D**) show median and upper/lower quartile, and whiskers extend to 1.5*IQR. **C**, **D** Data points represent 18 (V1), 28 (V2), and 21 (V3) cells. **E** Translocation was quantified by identifying the cell edge and defining a 10 pixel ring into the cytoplasm (“edge”). The remaining cell pixels beyond this ring wire designated as the cell “core”. Membrane localization was defined as the ratio of mean edge fluorescence to mean core fluorescence. Translocation was defined as the difference in adapter membrane localization after 1.5 min of EGF stimulation vs pre-stimulation. **F**, **G** Quantitation of translocation of GRB2 (**F**) or SOS (**G**) for cells transfected with one of the 3 EML4-ALK variants or for neighboring untransfected cells (wt). Due to small variations in imaging plane between acquisitions, the absolute magnitude of translocation differed between variants and drug conditions (note the differences in untransfected Beas2B responses, which are equivalent conditions between panels). However, cells with or without EML4-ALK (black vs. red in the same panel) were imaged in the same field of view and thus can be compared directly. Data points represent individual cells. For (**F**), *n* = 57(WT)/40(V1), 170(WT)/42(V2), 24(WT)/34(V3) cells. For (**G**), *n* = 46(WT)/50(V1), 50(WT)/27(V2), 67(WT)/40(V3) cells. Boxplots show median and upper/lower quartile, and whiskers extend to 1.5*IQR. **H** Definition of the magnitude of translocation. **I**, **J** Comparison of translocation suppression of GRB2 (**F**) or SOS1 (**G**) for each of the three variants. Data represent median translocation suppression from resampling of 1000 bootstrapped samples. Error bars show lower and upper quartiles. Significance determined by either one-sided T-test (panels **F**, **G**) or one-sided bootstrap test (panels **I**, **J**). Data for Variant 1 in (**F**–**J**) is reproduced from Fig. 4F, I. **K** Beas2B cells were transfected with EML4-ALK-2A-H2B-miRFP constructs for one of 3 EML4-ALK variants (V1, V2, V3), or with an H2B-iRFP control, and ppERK levels were assessed after stimulation with EGF (15 min, 50 ng/mL) in the presence or absence of ALKi (1 µM crizotinib, 2 h), through immunofluorescence. **L** Quantification of ppERK immunostaining after EGF stimulation of Beas2B transiently expressing EML4-ALK in the presence or absence of ALKi. Significance assessed by Hsu MCB test. *n* = 8 biological replicates. **M** ppERK response in the presence and absence of ALKi pretreatment. Data points represent the mean ppErk intensity of 20–60 cells. Significance assessed by one-sided T-test. *n* = 8 biological replicates. Gray bars in (**M**) are reproduced from (**L**) for direct comparison to between non-treated and ALKi-treated cells.

EML4-ALK(V1) is one of ~15 EML4-ALK variants found in human cancers^41^. Of these, variants 1,2 and 3 account for 70–90% of cases, with a roughly 3-fold higher frequency of V1 or V3 compared to the less-common V2^41^. EML4-ALK variants differ by the length of the EML4 fragment that is fused to the ALK kinase domain (Fig. 5A), and the differing lengths of EML4 are associated with different molecular interactions and condensation properties^20^. We thus asked whether EML4-ALK variants retain the ability to form multivalent assemblies, sequester GRB2 and SOS1 in the cytoplasm, and suppress RTK signal transduction, as observed in V1.

We expressed mCh-tagged fusions of each EML4-ALK variant in Beas2B cells where endogenous GRB2 or SOS1 was tagged with mNeonGreen2 (mNG2). All three variants could form cytoplasmic condensates (Fig. 5B, C, Supplementary Fig. S8A, C). On average, ~80–90% of EML4-ALK puncta overlapped with GRB2 puncta, and 60–90% visibly overlapped with SOS1 (Fig. 5D, Supplementary Fig. S8B, D). Treatment with ALKi (crizotinib, 1 µM) caused dissolution of GRB2 and SOS1 puncta across all variants (Supplementary Fig. S8E–H). Thus, each variant retained the ability to form multivalent assemblies of oncogene and adapters that depended on ALK kinase activity.

We next asked whether each variant could functionally sequester adapters during EGF stimulation by directly measuring cytoplasmic-to-membrane translocation of endogenous GRB2 and SOS1, as before (Fig. 5E). All variants suppressed translocation of both GRB2 and SOS1, with suppression strongest for V1 and V3 relative to V2 (Fig. 5F–J). Pre-incubation with ALKi relieved this suppression in all cases.

Suppression of adapter translocation corresponded to the ability of each variant to suppress EGF-induced ERK signaling (Fig. 5K–M). Expression of each variant increased ppERK levels relative to control cells, in line with the ability of each variant to drive oncogenic RAS/ERK signaling (Fig. 5L). However, in response to EGF, cells expressing EML4-ALK showed a markedly weaker response relative to controls (Fig. 5L). Signal suppression was stronger for V1 and V3 relative to V2, consistent with weaker suppression of adapter translocation for V2 (Fig. 5F–J). Pretreatment with ALKi reversed signal suppression for all variants, again with stronger reversal for V1 and V3 relative to V2, where initial suppression was milder (Fig. 5M).

In sum, this series of experiments reveals that, despite different sizes, structures, and biophysical properties of condensation^20^, three major EML4-ALK variants share the ability to form oncogene/adapter assemblies, sequester RTK adapters, and suppress transmembrane ligand-mediated signaling.

### Single-cell analysis of RTK resensitization reveals rapid signal reactivation after ALK inhibition

**Fig. 6:**
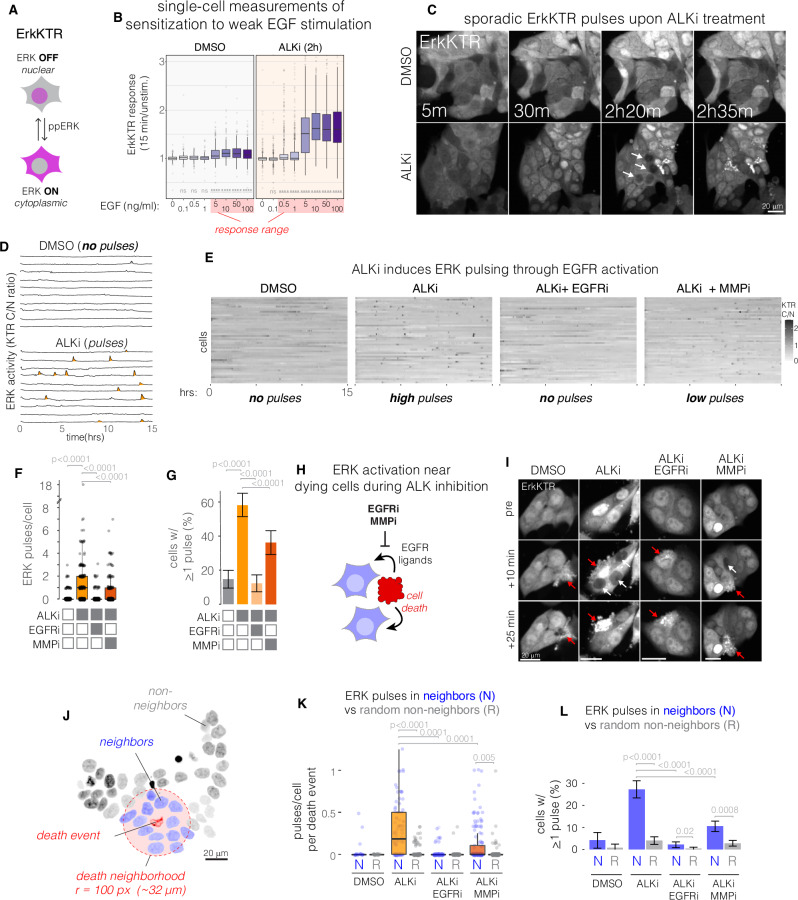
ALK inhibition hypersensitizes cancer cells to paracrine growth factors secreted from dying neighbor cells. **A** The ErkKTR reporter indicates ERK activity through nuclear-cytoplasmic translocation of a fluorescent protein. **B** Sensitivity of single cells (STE-1) to 15 min EGF stimulation. Plot shows fold change of ERK activity in single cells upon stimulation with the indicated amount of EGF in the presence or absence of ALKi (crizotinib, 1 µM). Boxplot shows median and upper/lower quartiles, and whiskers extend to 1.5*IQR. Significance tests indicate increased response above 0 ng/mL EGF. ****p < 0.0001 by Hsu multiple comparison with the best (MCB) test. *n* = 316, 384, 421, 285, 307, 256, 294, 421 cells respectively for DMSO and 473, 727, 536, 595, 541, 425, 439, 415 cells respectively for ALKi. See Supplementary Movie 3. **C** Live-cell imaging of STE-1 cells expressing ErkKTR in the presence or absence of ALKi (1 µM crizotinib). See Supplementary Movie 4. **D** Representative single-cell traces of cytoplasmic/nuclear ErkKTR intensity ratio from conditions shown in (**C**). **E** Quantification of ErkKTR activity in the presence of ALKi or its combination with EGFRi (1 µM erlotinib) or MMPi (10 µM marimastat). **F** Quantification of ERK activity pulses. Boxplot shows median and upper/lower quartiles, whiskers show 1.5*IQR. Significance assessed by Hsu MCB test. *n* = 177 (control), 198 (ALKi), 170 (ALKi/EGFRi), and 182 (ALKi/MMPi) single cells. **G** Percent of cells that exhibited any pulses over 22 h of imaging. Error bars indicate 95% CI. Significance assessed by the Hsu MCB test. *n* = 200 cells per condition. **H** Apoptotic cells secrete paracrine EGFR ligands to their neighbors. Paracrine signaling can be blocked by inhibiting either EGFR or the MMPs that mediate shedding of EGFR ligands from the surface of the sender cell. **I** ErkKTR activity pulses are primarily observed surrounding a dying cell during ALK inhibition but not in the absence of drug or in the added presence of EGFR or MMP inhibitors. **J** Definition of neighbors and non-neighbors of a death event. **K** Quantification of pulses per cell for each death event in neighbors or in equal number of randomly chosen subset of cells not near a death event (see “Methods” for more details), *n* = 91, 103, 149, 20 events for ALKi, ALKi/EGFRi, ALKi/MMPi and DMSO, respectively. Boxplot shows median and upper/lower quartiles, whiskers show 1.5*IQR. **L** Fraction of total neighbor vs random non-neighbor cells that show any pulsing. Cell numbers as in (**F**). Error bars show 95% CI. Significance in (**K**, **L**) determined by independent T-tests (within treatment conditions) or by ANOVA followed by the Hsu MCB test (across treatment conditions).

RTK activity promotes drug tolerance across cancer types^15,28^. We hypothesized that drug-dependent resensitization of RTKs could promote RTK signaling during ALKi therapy. We thus monitored ERK signaling in drug-treated populations of STE-1 cells using ErkKTR, a biosensor that reports on ERK activity through nuclear exclusion of a fluorescent protein^42^ (Fig. 6A). We first used ErkKTR to confirm that ALKi sensitized cells to EGF ligands, finding that ALKi-treated cells showed measurable increase in ERK activity at ~10-fold lower EGF concentrations than untreated cells (Fig. 6B, Supplementary Fig. S9A, Supplementary Movie 3). Moreover, at any concentration above this response threshold, the magnitude of single-cell responses was uniformly stronger in the presence of ALKi (Supplementary Fig. S9B, Supplementary Movie 3), paralleling our earlier results in fixed cells.

We then observed ERK activity after ALKi treatment in the absence of exogenous stimulation. In untreated STE-1 cells, ERK activity was at an intermediate level, indicated by relatively equal reporter distribution between the cytoplasm and nucleus (Fig. 6C, top row, Supplementary Movie 4). Notably, this localization pattern did not change over the course of 22 h, reflecting the tonic signaling downstream of EML4-ALK. As anticipated, treatment with ALKi induced a rapid initial decrease of ERK activity (Fig. 6C, bottom, 5 m and 30 m panels). Strikingly, this initial decrease was followed by the appearance of sporadic ERK activity pulses within ~1–2 h of treatment (Fig. 6C, 2h20m panel, Supplementary Movie 4). Each ERK pulse lasted 10–20 min, and the pulse amplitude exceeded ERK activity levels observed in the absence of drug (Fig. 6D).

Activity pulses appeared sporadically but in a spatially coordinated manner, appearing either simultaneously or as a traveling wave within small clusters of neighboring cells (Fig. 6C, Supplementary Movie 4). This pattern was consistent with RTK stimulation through paracrine signaling. To test this hypothesis, we sought to block paracrine signals through inhibition of either the EGF receptor (EGFRi, 1 µM erlotinib) or matrix metalloproteases (MMPi, 10 µM marimastat), which release EGFR ligands from the cell surface to enable paracrine signaling^43–45^. Co-treatment with ALKi and EGFRi eliminated ERK pulses after drug addition, indicating that EGFR activation causes the observed ERK pulses (Fig. 6E–G, Supplementary Movie 4). Similarly, co-treatment with ALKi and MMPi also reduced ERK reactivation pulses, though to a lesser extent than with EGFRi, potentially due to MMP-independent juxtacrine signals^46,47^(Fig. 6E–G, Supplementary Movie 4). Thus, ALKi treatment decreases ERK activity but is rapidly followed by RTK reactivation mediated by paracrine signals.

### Signal reactivation results from paracrine signals from dying cells

We next sought to determine the source of the paracrine signals. We observed that pulsing events appeared next to dying cells and that co-inhibition of ALKi with EGFRi or MMPi prevented this pulsing (Fig. 6H, I, Supplementary Movie 4). These observations are consistent with paracrine ligand secretion from apoptotic cells, which promotes survival of neighboring cells and homeostasis of epithelial sheets^48–50^. To quantify this effect, we measured signal activation in cells that neighbored a dying cell within the ~hour preceding its death (Fig. 6J). We then counted ERK pulses in these neighbors (N) and compared pulse counts to those from randomly selected non-neighbors (R) over that same time interval (for more details, see “Methods”). In untreated cells, pulses were almost never observed in either the N or R populations (DMSO, Fig. 6K, L), demonstrating the need for ALK inhibition for RTK/Erk activation. However, in ALKi-treated cells, N cells pulsed significantly more than R cells (Fig. 6L). Co-treatment with either EGFRi or MMPi dramatically reduced ERK pulsing in neighbors (Fig. 6L). Ligand shedding during apoptosis is triggered by mitochondrial outer membrane permeabilization (MOMP) and thus cannot be blocked by inhibition of apoptotic caspase cleavage, which occurs downstream of MOMP^48^. Accordingly, co-treatment of cells with ALKi and a caspase inhibitor suppressed apoptosis but did not suppress ERK pulsing, in line with MOMP-induced ligand shedding (Supplementary Fig. S9C–E, Supplementary Movie 5). Together, our results demonstrate that virtually all observed ERK reactivation is associated with paracrine signals associated with dying drug-treated cells. Importantly, because ERK pulses were not observed in untreated cells—even in neighbors of dying cells (Fig. 6I, K, L)—ALKi-induced RTK resensitization is an essential first step for the perception of paracrine ligands during ALKi therapy.

### Signal reactivation pulses activate downstream gene expression

**Fig. 7:**
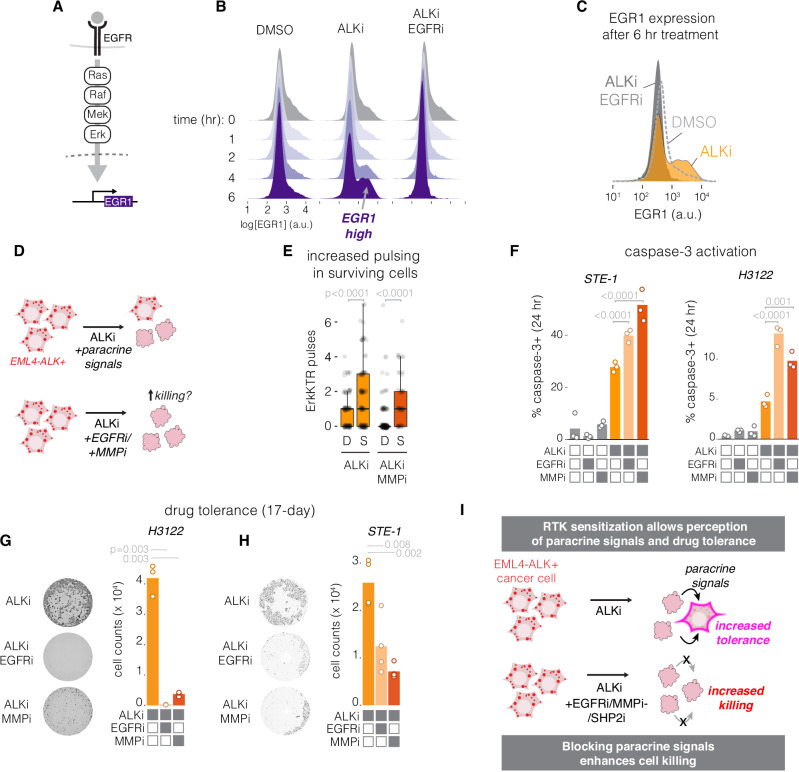
Resensitization to paracrine signals drives gene expression and promotes drug tolerance. **A** Signaling through EGFR activates RAS/ERK and stimulates transcription, including of the immediate early gene EGR1. **B** Quantification of single-cell IF of EGR1 in STE-1 cells under the conditions indicated. **C** Overlay of EGR1 expression at the 6 h time point in (**B**). **D** Testing whether restored perception of paracrine signals can promote survival during ALKi treatment. **E** Quantification of pulses per cell in cells that died (*D*) or survived (*S*) through 22 h of imaging in the conditions where ERK pulsing could be observed. Boxplot shows median and upper/lower quartiles, whiskers show 1.5*IQR. Significance assessed by one-sided T-tests. *n* = 88 ALKi(D), 110 ALK(S), 136 ALKi/MMPi(D), and 46 ALKi/MMPi(S) cells. **F** Caspase-3 activation was assessed using the NucView reporter after 24 h treatment with the indicated drugs. Data points show proportion of 2300–3500 STE-1 cells and 3500–4500 H3122 cells. Significance assessed by one-sided T-test, *n* = 3 biological replicates. **G**, **H** DAPI imaging (left) and cell counts (right) of cell survival after 17 days of the indicated treatments in both H3122 (**G**) and STE-1 (**H**) cell lines. Significance assessed using one-sided T-test. *n* = 3 (H3122) and *n* = 4 (STE1) biological replicates. **I** Summary of the effects of drug-induced RTK resensitization.

We next asked whether the short ALKi-dependent ERK pulses could impact cell behavior. We first asked whether ERK pulses led to downstream transcription. ERK activity controls the transcription of immediate early genes (IEGs) which begin transcription within minutes of ERK activity^51^. EGR1 is an IEG that has been implicated in drug resistance to ALK inhibitors^16^. Additionally, EGR1 expression is adaptive, such that its expression peaks by ~1 h but then decays within 1–2 h, even in the presence of constant upstream signal^29,52^ (Supplementary Fig. S10A, B). Thus, the accumulation of EGR1 indicates the presence of only recent ERK activation^53^. We examined the extent to which EGR1 accumulated in STE-1 cells upon drug treatment to understand whether drug-induced ERK pulses could drive transcription (Fig. 7A). In untreated cancer cells, EGR1 levels remained low despite high ERK signaling from EML4-ALK, consistent with EGR1 adaptation to tonic ERK signals (Fig. 7B, C). By contrast, ALK inhibition resulted in a distinct peak of EGR1-high cells that appeared 4 h after drug treatment. Co-inhibition of ALK and EGFR prevented the appearance of EGR1-positive cells, consistent with transcription resulting from paracrine signaling through EGFR (Fig. 7B, C, Supplementary Fig. S10C). Similar responses were measured in H3122 cells as well as in two additional primary patient-derived EML4-ALK(V1)+ cell lines (Supplementary Fig. S10D–I). Thus, ALKi-induced ERK activity pulses provide sufficient signal to drive gene expression changes that could regulate cell fate.

#### Signal reactivation promotes acute drug tolerance and cell persistence during ALK inhibition

Finally, we asked whether ALKi-induced RTK resensitization and ERK pulses could counteract cell killing and promote tolerance to ALKi therapy (Fig. 7D). First, we observed that cells that survived over the first 22 h of treatment showed more ERK pulses compared with those that died (Fig. 7E, Supplementary Fig. S11A). To quantify cell death, we measured a fluorescent reporter of caspase-3 activity (NucView) after the first 24 h of drug treatment. While ALKi monotherapy led to increased caspase-3 over baseline, co-treatment with either EGFRi or MMPi significantly increased the caspase-3+ cell fraction in both STE-1 cells and H3122 cells (Fig. 7F, Supplementary Fig. S11B). Neither EGFRi nor MMPi treatment alone showed substantial killing over untreated cells. Enhanced killing was also observed when alectinib was used as the ALK inhibitor in combination with either EGFRi or MMPi (Supplementary Fig. S11C). Conversely, addition of 50 ng/mL EGF promoted survival in the presence of ALKi/MMPi in STE-1 cells (Supplementary Fig. S11D). Similarly, optogenetic pulses of optoFGFR lowered cell death in cells treated with combined ALKi/EGFRi treatment (Supplementary Fig. S11E–G). Finally, co-treatments of ALKi with either EGFRi or MMPi suppressed drug tolerance measured at 17 days in both STE-1 and H3122 cell lines (Fig. 7G, H), consistent with prior reports that demonstrated the anti-tumor efficacy of ALKi/EGFRi co-treatment in cell and xenograft models^13,14,16,54^.

Our mechanistic studies also suggested that other clinically relevant drug combinations would suppress ERK reactivation and cancer cell survival. SHP2 is an intracellular phosphatase involved in transducing RTK signals^55,56^ and co-therapies with SHP2 inhibitors can enhance response to ALK inhibitors^57,58^. Paralleling our results with EGFRi and MMPi, SHP2i co-treatment with ALKi suppressed RTK reactivation pulses and enhanced cell killing, while SHP2i alone had little effect (Supplementary Fig. S12A–C).

Together, our results indicate that ALK inhibitors resensitize transmembrane EGFR and restore perception of paracrine ligands. Resensitized EGFR allows reactivation of survival signaling within minutes of drug treatment, which limits the cytotoxicity of ALK therapies and promotes drug tolerance, the first step towards acquired resistance (Fig. 7I). The molecular mechanisms we find to underlie these events likely contribute to previously observed synergies in multiple combination therapies and propose treatment co-targets to enhance therapy in EML4-ALK+ cancers.

## Discussion

Our results reveal an apparently paradoxical function for EML4-ALK assemblies in cancer cells and their response to targeted therapy^59^. Cytoplasmic assemblies both facilitate oncogenic ALK signaling^19^ and simultaneously suppress transmembrane RTK signaling by acting as molecular sponges for shared adapters including GRB2 and SOS1. This sequestration desensitizes the cell’s perception of external ligands. However, upon ALK inhibition, adapters are rapidly released as the oncogene is silenced, resensitizing the cellular response to ligands in the cell’s microenvironment. This resensitization counteracts cell killing in response to targeted inhibitors, due at least in part to death-induced paracrine signals.

Adapter sequestration suppresses at least two key features of RTK signal transmission: both the maximal ERK signal amplitude (Figs. 1F, 2B, S9A) as well as the fold-change of response (Figs. 1G, 2B, 2D, 6B, S9B). While the relative importance of these features is not yet clear, fold-change detection has been proposed as a central feature of multiple signaling pathways, including within EGFR/ERK signaling^60–62^. Fold-change detection permits a high-resolution perception of the cellular environment over multiple orders of magnitude of ligand concentration. EML4-ALK activity compressed the dynamic range of response to a 10-fold change in EGF concentration (1–10 ng/mL) (Fig. 6B, Supplementary Fig. S9B), and ALK inhibition restores this response by at least an order of magnitude. Further work will determine whether drug treatment restores a normal perceptive state or alternatively represents a hyper-perceptive state that could present additional therapeutic vulnerabilities.

EGFR signaling promotes survival and resistance to ALK inhibitors in EML4-ALK+ cancer cells in vitro, in animal studies, and in patients^13–15,17,25,27,54^. Our work reveals cell-intrinsic and -extrinsic mechanisms that promote rapid EGFR reactivation after drug treatment. These likely act in concert with previously described cell-intrinsic mechanisms that act on longer timescales, including amplification of KRAS and downregulation of DUSP6^8^, autocrine EGFR signaling^63^, and induction of stress responses downstream of sublethal cytochrome c release^64^. Despite the efficacy of ALKi/EGFRi co-therapy in vitro and in vivo^13,14,16,54^, this combination failed in a recent clinical trial due to frequent adverse effects and low maximum tolerated dose^65^. Nevertheless, our mechanistic studies suggest other drug co-targets to enhance response to ALK inhibitors. One such co-target is SHP2. This combination is effective in mouse xenografts^57,58^ and is currently under evaluation in clinical trials (ID: NCT04292119). Another possible co-target is MMPs, which are required for ligand shedding from apoptotic cells. While this combination has not yet been tested, previous trials using high concentration of MMP inhibitors like marimastat have suffered from dose-limiting toxicities^66–68^, and additionally long-term MMP inhibition can promote resistance by causing accumulation of transmembrane RTKs (e.g., AXL) that are also MMP targets^69^. Careful studies will be required to understand whether MMPi co-treatment can indeed enhance therapy, or whether MMPi scheduling could leverage acute benefits while avoiding deleterious chronic effects. More broadly, the mechanisms we uncover may inspire new types of therapies that recapitulate adapter sequestration as a means to suppress RTK reactivation. Of note, the recent discovery of EML4-ALK condensates has raised the idea that disaggregation of the condensates might be therapeutically beneficial^70,71^. Our work cautions, however, that such disaggregation strategies will likely be subject to the same adapter redistribution and rapid RTK reactivation observed with small molecule ALK inhibitors.

Although our study focused on EML4-ALK(V1), we find that modulation of transmembrane RTK sensitivity may be a widespread feature of RTK fusions. Three distinct cancer-associated EML4-ALK variants (V1, V2, V3) can form condensates (Fig. 5B, C, Supplementary Fig. S8A, C), sequester adapters (Fig. 5E–J), and suppress RTK signaling (Fig. 5L, M), regardless of differences in EML4 length, propensity for condensation, and biophysical properties (e.g., solid vs liquid-like)^19,20^. Additionally, cancer cells driven by a CCDC6-RET, a distinct fusion shown to form condensates, also showed EGFR suppression that was restored upon drug treatment (Supplementary Fig. S7A, B). Because multivalency and RTK adapter recruitment are common motifs of RTK fusions, the molecular mechanisms we describe may act across this large class of oncogenes^19,72^. Indeed, a recent study found drug-induced association of GRB2 with EGFR in multiple cancers harboring fusions of ALK, RET, NTRK1 and ROS1, including in patient samples of primary tumors, resistant tumors, and metastases^13^. Interestingly, we found no evidence of adapter sequestration and signal suppression in cells harboring transmembrane mutant RTKs (Fig. 2F, Supplementary Fig. S7C, D), possibly because normal and mutant transmembrane RTKs share the membrane compartment, or because transmembrane receptors form smaller or weaker assemblies compared to the cytoplasmic fusions.

Although our study highlights how cytoplasmic assemblies can functionally sequester adapters from the membrane, it does not indicate whether assemblies require certain sizes or biophysical properties for this effect. For example, although large condensates of EML4-ALK are readily apparent under a microscope, condensates can also form at submicroscopic length scales that are not readily observable due to limitations of conventional microscopy^73^. Further studies will inform how condensate size is controlled and the effect of size on sequestration.

A unique promise of functional profiling of cancer cells is that common network-level signaling abnormalities may be identified and inform therapies among molecularly distinct cancers^30^. Oncogene-induced suppression of RTKs is one such abnormality that has been observed previously in BRAF V600E+ melanoma and colorectal cancer cells, though through distinct mechanisms to the ones we report^9–11^. Inhibition of BRAF suppressed these mechanisms and led to rapid, pulsatile ERK reactivation and drug resistance in vitro and in vivo. Separately, in cancers driven by EGFR, ERK provides suppressive phosphorylation of EGFR receptors, which is lost during MEK inhibition and leads to reactivation of ErbB3 and PI3K^74^. Our findings show that RTK suppression can be implemented not just through biochemical feedback, but also through biophysical feedback mediated by sequestration of adapters within RTK fusion assemblies. The variety of mechanisms by which oncogenes can suppress RTKs hints that such feedback might be important to establish permissive conditions for oncogenesis, and that relief of this feedback must be considered to achieve durable therapy. It will thus be important to more comprehensively understand the diverse oncogenic contexts in which such suppression and reactivation occurs.

## Methods

### Cell lines and cell culture

All cell lines were maintained at 37 °C and 5% CO_2_ using a standard cell culture incubator. STE-1, H3122, TPC-1, Beas2B, TE-6, TE-8, TE-11, NCI-N87, HCC827, CUTO-8 and CUTO-9 cells were cultured in RPMI-1640 growth medium supplemented with 10% fetal bovine serum (FBS) and 1% penicillin/streptomycin (P/S). SH-SY5Y, KELLY and LentiX- HEK 293T cells (TakaraBio, #632180) were cultured using DMEM supplemented with 10% FBS and 1% P/S. For experiments, cells were seeded in 96 or 384-well plates coated with fibronectin (MilliporeSigma, FC01010MG) diluted to 10 µg/mL in PBS. STE-1, H3122, TPC-1, CUTO-8, SH-SY5Y, and Kelly cells were seeded in 96-well plates at 10^4^ cells or in 384-well plates at 5 × 10^3^ cells per well. The rest of the cell lines were seeded at 5 × 10^3^ (96-well) or 1.5 × 10^3^ (384-well) cells per well. Unless indicated otherwise, cells were serum-starved overnight before experiments by performing multiple washes with serum-free medium, either manually or using the BioTek 405 LS microplate washer. Beas2B cells expressing endogenously tagged *GRB2:mNG* (Beas2B GRB2:mNG) were generated previously^19^. CUTO-8 and CUTO-9 cell lines were derived from patient tumors^75,76^. SH-SY5Y and KELLY were generous gifts from Dr. Arjun Raj. HCC827 was a generous gift from Dr. Sydney Shaffer.

### Reagents and inhibitors

Unless indicated otherwise, cells were pre-treated for 2 h with all inhibitors: crizotinib (1 µM, Sigma-Aldrich, PZ0191), alectinib (1 µM, Selleckchem, CH5424802), lapatinib (10 µM, Tocris, 6811), erlotinib (1 µM in EML4-ALK driven cell lines or 2 µM in EGFR driven cell lines, Selleckchem, OSI-774), marimastat (10 µM, Selleckchem BB-2516), RMC-4550 (0.5 µM, Cayman Chemical, 31011-1), Z-VAD-FMK (50 nM, Selleckchem, S7023) BLU-667 (0.1 µM, Biovision, B2548). Cells were stimulated with EGF (50 ng/mL unless stated otherwise, PeproTech, 315-09) or HGF (50 ng/mL, R&D 294-HG). Cells expressing iRFP fluorophore were treated with biliverdin (10 µM, Cayman Chemicals, 19257) to allow for quantitative comparison between cells^77^. Caspase-3 activity was detected by treating cells with NucView488 (1 µM, Biotium, 10402) along with the respective inhibitor.

### Plasmid design and assembly

All cloning was performed by PCR and DNA assembly using NEBuilder® HiFi DNA Assembly Master Mix (New England Biolabs #E2621) or using blunt end ligation using NEB T4 ligase (NEB, #M0202). For live-cell tracking of ERK activity, we generated pHR ErkKTR-mRuby2 by inserting an mRuby2 coding sequence in place of BFP in a construct we described previously^34^. Visualization of nuclei was achieved using pLentiPGK DEST-H2B-iRFP670 (Addgene:#90237). For GRB2 overexpression experiments, GFP or GRB2-GFP was amplified from (Addgene: #86873) and cloned into a CLPIT or pEGFP-C1 backbone. For SOS1 overexpression, SOS1 was amplified from (Addgene: #32920) and cloned into a pCMV backbone upstream of P2A-H2B-mRuby2. A pCMV-H2B-mRuby2 control construct was generated from this same backbone. EML4-ALK(V1) was obtained from a previously described construct^8^. To construct EML4-ALK-P2A-H2B-iRFP (pCMV EML4-ALK-P2A-H2B-iRFP), H2B-iRFP was amplified from pLentiPGK DEST-H2B-iRFP670 with P2A encoded as an overhang on the 5’ primer and was fused to the 3’ end (C terminus) of EML4-ALK(V1) in a pCMV vector. EML4-ALK V2 or V3 were cloned by inserting or excising the relevant domains according to the following coding sequences: EML4-ALK V2, GenBank: AB275889; EML4-ALK V3, GenBank: AB374361.1^78^. All EML4-ALK variants were also cloned downstream of mCherry in a pCMV-mCh plasmid. EML4-ALK (∆TD) mutant was cloned by removing residues 310–459 from pCMV-EML4-ALK-P2A-H2B-iRFP. EML4-ALK (K589M) was cloned by single point mutation encoded on the primer^78,79^. Constructs for generation of stable cell lines were cloned into pHR lentiviral or CLPIT retroviral plasmid backbones. For optogenetic control of FGFR signaling^31^, the FGFR intracellular domain with an N-terminal myristoylation site was inserted upstream of mCh-Cry2^80^ in a CLPIT plasmid to create CLPIT Myr-mCh-FGFR(ICD)-Cry2. For optogenetic control of SOS signaling, we generated pHR sspB-SOScat-mCh-2A-iLid-CAAX as described previously^34,81^.

### Transient transfection and generation of stable cell lines

Beas2B cells were transfected using Lipofectamine™ 3000 (Invitrogen, L3000001) according to the manufacturer’s protocol. Briefly, 1500 cells were seeded in fibronectin-coated wells of a 384-well plate in RPMI growth medium. Each well was supplemented with 25 ng of DNA plasmid, 0.5 µL of P3000 reagent and 0.25 µL Lipofectamine, diluted in OptiMEM (Gibco, #31985070). H3122 cells were transfected using Fugene4K (Promega, E5911). Then, 5000 cells were seeded in fibronectin-coated wells of a 384-well plate in RPMI growth medium. Each well was supplemented with 25 ng DNA plasmid and 0.75 µL of Fugene4K reagent diluted in OptiMEM.

Lentivirus was produced 7 × 10^5^ HEK 293T cells transfected with 1.5 µg of transfer plasmid 1.33 µg of pCMV-dR8.91 (Addgene #12263), and 0.17 µg pMD2.G (Addgene #12259). For CLPIT transfer vectors, cells were transfected with 1.25 µg of transfer vector, 0.5 µg of pCMV-VSVG (Addgene #8454) and 0.75 µg of pCMV-gag/pol. Transfections were performed using the calcium phosphate method. Virus-containing supernatant was collected 48 and 72 h post-transfection and filtered through a 0.45 µm filter (Fisher Scientific, #13-1001-07). To generate stably expressing cell lines, transduced cells were expanded and sorted (BD FACS ARIA Fusion) for appropriate expression levels. CLPIT-infected cells were selected with puromycin (0.5 µg/mL, RPL).

### Optogenetic stimulation

Light stimulation was achieved in microwell plates using the optoPlate-96 (1-color blue version)^82^. A 20 mm tall black adapter was used to ensure even light diffusion across each of the 384-well plate wells. Cells were stimulated with 500 ms every 10 s (light intensity ranging from 3.2 mW/cm^2^ (min) to 160 mW/cm^2^ (max)). For optogenetic rescue of cell death during ALKi/EGFRi treatment, cells were stimulated as described with blue light for 10 min every hour, with stimulation beginning simultaneously with drug treatment. All media changes and reagent supplementations were performed in the dark.

### Growth factor stimulation assays

Unless otherwise indicated, starved cells were treated with the indicated inhibitors for 2 h and then stimulated at their respective time points with EGF or HGF. Cells were then fixed simultaneously for 10 min with 4% paraformaldehyde (PFA) (Electron Microscopy Sciences, 15710). Samples were then processed for immunostaining.

### Immunostaining

Fixed cells were permeabilized with 0.5% Triton X-100 in PBS for 10 min, followed by incubation in ice-cold 100% methanol for 10 min. Samples were then blocked with blocking solution (1% bovine serum albumin (BSA, Fisher, BP9706100) diluted in PBS) for 1 h at room temperature. Samples were incubated in indicated primary antibody diluted in blocking solution for either 2 h at room temperature (RT) or overnight at 4 °C. Primary antibodies used were: phospho-p44/42 MAPK (ERK1/2) (Thr202/Tyr204), Cell Signaling #4370; phospho-EGF Receptor (Tyr1068), Cell Signaling #3777; phospho-ALK receptor (Tyr 1507), Cell Signaling #14678; EGR1, Cell Signaling #4153. Antibodies were used at dilutions recommended by the manufacturer. After incubation with primary antibody, samples were washed 7X with 80% washes of 0.1% Tween-20 in PBS (PBS-T) using the BioTek 405 LS microplate washer. Samples were then incubated in blocking solution containing secondary antibody IgG (H+L) Cross-Adsorbed Goat anti-Rabbit, DyLight™ 488, Invitrogen #35553; Goat anti-Rabbit IgG (H+L) Cross-Adsorbed Secondary Antibody, DyLight™ 650, Invitrogen #SA510034; and 4,6-diamidino-2-phenylindole (DAPI; ThermoFisher Scientific #D1306, 300 nM) for 1 h at RT. Samples were washed with PBS-T as previously described.

### Immunoblotting and co-immunoprecipitation

For immunoblots, STE1 or H3122 cells (3 × 10^5^) were plated in each well of a 6-well plate, cultured for 24 h, and subsequently serum-starved for 16 h. Cells were treated with crizotinib for the times indicated, after which cells were washed in ice-cold PBS and lysed in RIPA buffer (50 mM Tris pH 7.5, 150 mM NaCl, 1% NP40, 0.1% SDS, 0.5% DOC, 1 mM EDTA, 2 mM sodium vanadate and protease inhibitor (Sigma #P8340)). Protein concentration of cleared cell lysates was determined by BCA protein assay kit (Thermo Scientific #23225) and 30 µg of lysed samples were subjected to SDS–polyacrylamide gel electrophoresis (SDS-PAGE). For co-immunoprecipitation, STE1 or H3122 cells (2.5 × 10^6^) were plated on 10 cm plates, cultured for 24 h, and subsequently serum-starved for 16 h. Cells were pre-treated with crizotinib for 20 min and stimulated with 20 ng/mL EGF for 2 min, washed with ice-cold PBS, and lysed (50 mM HEPES pH7.4, 150 mM NaCl, 1% Triton X-100, 1 mM EDTA, 1 mM EGTA, 10% glycerol, 2 mM sodium vanadate and protease inhibitor). Cleared cell lysates were incubated for 2 h with Protein A/G agarose beads (Santa Cruz, SC-2003) that were hybridized with EGFR antibody (Thermo, clone H11). Beads were then washed 5 times with HNTG buffer (20 mM HEPES pH 7.4, 150 mM NaCl, 0.1% Triton X-100, 10% glycerol), and sample buffer was added to elute proteins. Eluates or 30 μg of whole cell protein lysate were loaded in a precast 4–15% gradient SDS–polyacrylamide gel for electrophoresis (mini-protean TGX precast gel, Bio-RAD, #456-1084).

Protein separations were transferred onto a nitrocellulose membrane using the Trans-blot Turbo RTA transfer kit (Bio-rad, #170-4270) according to the manufacturer’s protocol. Membranes were blocked in 5% milk in Tris buffer saline with 0.5% Tween-20 (TBS-T) for 1 h and incubated overnight at 4 °C with primary antibodies against EGFR (CST #4267), SOS1 (CST #5890), SPRY2 (CST #14954), pERK1/2 (CST #4370), ERK (CST #4695), ALK (CST #3633) tubulin (CST #3873) or GRB2 (Thermo Fisher Scientific, PA1-10033). Each primary antibody was used at a dilution of 1:1000 in TBS-T with 3% BSA. After washing with TBS-T, membranes were incubated with secondary antibodies in TBS-T with 3% BSA for 1 h at room temperature (IRDye® 800CW Goat anti-Rabbit IgG, LI-COR #926-32211; IRDye® 680RD Donkey anti-Mouse IgG, LI-COR, #926-68072; Alexa Fluor® 790 AffiniPure Donkey Anti-Rabbit IgG (H+L) Jackson #711-655-152). Membranes were then imaged on the LI-COR Odyssey scanner. Densitometry was performed using ImageJ.

### Live-cell imaging

Live-cell imaging was performed using a Nikon Ti2-E microscope equipped with a Yokagawa CSU-W1 spinning disk, 405/488/561/640 nm laser lines, an sCMOS camera (Photometrics), and a motorized stage. Cells were maintained at 37 °C and 5% CO_2_ using an environmental chamber (Okolabs). All imaged wells were cultured in phenol-free RPMI. For adapter localization assay in Beas2B *GRB2:mNG2* or *SOS1:mNG2* cells, cells were serum-starved before imaging. For imaging of adapter localization during EML4-ALK inhibition, cells were imaged every 5 min. Adapter translocation in response to EGF stimulation was imaged every 30 s using a 40× oil-immersion objective. For colocalization of EML4-ALK and GRB2/SOS1, the appropriate Beas2B cell line was imaged in RPMI growth media using an Olympus IX71 inverted microscope with 40× air objective. For monitoring ERK activity after drug treatment, STE-1 cells stably expressing ErkKTR-mRuby2 and H2B-iRFP670 were seeded in a 96-well plate (Falcon #353072) and cultured in 2% FBS and 1% P/S. Immediately before imaging, cells were supplemented with NucView (Biotium #10402, 1 µM) and the indicated drugs. Cells were imaged every 5 min using a 20× magnification objective.

### Caspase-3 activation and drug tolerance assay

To assay acute cell killing, STE-I and H3122 cells were seeded in a 96-well plate. The following day, media was replaced with phenol-free RPMI-1640 supplemented with 2% FBS, 1% P/S, and treated with 1 µM NucView 488 and the indicated inhibitors. 24 h after drug addition, cells were incubated in 5 µg/mL Hoechst 33342 Hydrochloride (Cayman 15547) for 15 min. Cells were then imaged under live-cell confocal microscopy. When inhibitor treatment was performed for 5 days, 5 µg/mL Hoechst  was added to each well 15 min before imaging as described. To assay drug tolerance, cells were treated as described and cultured for 17 days, with fresh media exchanges every 2 days. Cells were fixed with 4% PFA, permeabilized with 100% methanol for 10 min, incubated in DAPI (300 nM) for 30 min, imaged, and nuclei were counted.

### Image processing and analysis

Images of fixed-cell immunostaining and live-cell caspase-3 reporter were quantified using CellProfiler (v 4.0.7)^83^. Briefly, cell nuclei were segmented using the DAPI channel, and the cytoplasmic fluorescence was measured within a 5-pixel ring that circumscribed the nucleus. For measuring caspase-3 activity and drug tolerance, DAPI or Hoechst-stained nuclei were segmented using ilastik^84^, quantified in CellProfiler, and quantification was exported to R for processing and data visualization using the tidyR package^85^.

### GRB2 and SOS1 localization

Time-lapse imaging of GRB2 and SOS1 puncta was quantified with a custom MATLAB script. Briefly, cells were segmented manually and clusters were identified in a semi-automated manner through the following steps: First, cell intensities were normalized to their internal median. Images were then passed through sequential Gaussian, top hat, and Laplacian filters to enhance clusters while suppressing other features like background and high-frequency noise. These transformed intensities were used to identify bright pixels at the center of clusters with a user-defined intensity cutoff. From each of these center points, neighboring pixels were compared to a threshold intensity that was set by the local background of the cell and scaled by a second user-defined parameter. Neighboring pixels above this threshold are included in the cluster, while pixels below the threshold are excluded. This occurs iteratively, where adjacent pixels to each included pixel were also checked to be included or excluded until all new neighbors were below the threshold. Cluster properties of each cell were exported and processed in R for analysis and visualization. The full analysis pipeline can be found on Github: https://github.com/BugajLab/Cluster-Fitting.

### ErkKTR activity

ErkKTR dynamics and cell death were tracked and quantitated in a semi-automated manner using the p53CellCinema package^86^ in MATLAB, and data was processed and visualized in R and RStudio using the tidyR package. Briefly, following the tracking of at least 250 cells per frame, cells with low or no ErkKTR expression were first excluded from the analysis. Then, ErkKTR cytoplasmic/nuclear ratios from the remaining cells were imported into MATLAB, and peaks were identified using the ‘findpeaks’ function. Peak calls were then manually inspected for outliers, for example resulting from apoptotic cell debris, and outlier cells were removed from analysis. Of note, we consistently observed ERK pulses preceding cell division events across conditions. This phenomenon has been previously observed and has been shown to be independent of RAS/ERK signaling, potentially due to non-specific activation from a cyclin-dependent kinase^11,87^. Thus, we disregarded all pulses that occurred within 10 frames (50 min) preceding cell division. To count ERK pulses in neighbors vs non-neighbors of dying cells, we first identified each cell death event. We then identified the neighbors of the dying cell by identifying which nuclear centroids were within a 100-pixel radius (~32 µm, or 2–3 cell diameters) of the dying cell for each of the 10 frames preceding the death event. We then counted the total pulses of those neighbors over the indicated time frame and compared against pulse counts from a random subset of non-neighbor cells over the same time frame. Importantly, for each death event, the number of non-neighbors matched the number of neighbors, and the sampled non-neighbors were not neighbors of other death events over that same time span. For visualization in Fig. 6E, cells that divided during the course of the experiment are represented as separate cells, and the ERK history of the parent cell is reproduced for both cells. However, for quantification of ERK pulses, parent cell pulses were counted only once.

### GRB2 overexpression studies

To study the effects of adapter overexpression, we generated stable cell lines that expressed either GRB2-GFP or GFP alone using lentiviral transduction and puromycin selection. It was important to compare signaling between cells expressing GRB2-GFP vs GFP alone as a function of concentration of the transgene because we found that overexpression itself (e.g., of GFP alone) could alter the EGF-induced ppERK responses at high expression levels. Similarly, for SOS1 overexpression in H3122 cells (Supplementary Fig. S5J), comparisons were made between H3122 cells (stably expressing either GFP or GRB2-GFP) that were transfected with SOS1-P2A-H2B-mRuby2 construct or a H2B-mRuby2 control plasmid, and both GFP and mRuby2 fluorescence were used to ensure comparison between the same levels of the respective transgenes.

### Statistics

The Kolmogorov-Smirnov (KS) test was used for comparison of two distributions. Means between two groups were compared using either T- or Z-tests. Medians were compared by the nonparametric Wilcoxon test when normality of the data could not be assumed. Three- or four-group mean comparisons were made using ANOVA, followed by multiple comparison methods: either the Hsu test for comparison with the maximum/minimum mean^88^, or the Student’s T-test for comparison of each pair. Medians of the index of suppression (Figs. 4I, 5I, J) were compared by a bootstrap test. Analyses were performed using JMP® Pro, version 17.0.0, and R, version 4.0.3.

### Reporting summary

Further information on research design is available in the Nature Portfolio Reporting Summary linked to this article.

## Supplementary information

Supplementary Information

Movie S1

Movie S2

Movie S3

Movie S4

Movie S5

Description of Additional Supplementary Files

Peer Review File

Reporting Summary

## Source data

Source Data

## Data Availability

Data generated from this study are available within the article, Supplementary Information or Source Data file. Source data are provided with this paper.

## Code availability

MATLAB code used to analyze cluster number, area, and colocalization can be found on Github: https://github.com/BugajLab/Cluster-Fitting. Custom scripts used to analyze all immunofluorescence data are available upon request.
