# Supplementary material for: Oncogenic EML4-ALK assemblies suppress growth factor perception and modulate drug tolerance
Source: Nat Commun. 2024 Nov 2;15:9473. doi: 10.1038/s41467-024-53451-7 (PMC11531495; doi:10.1038/s41467-024-53451-7)
Supplement: Supplementary file 1 — Supplementary Information [file 41467_2024_53451_MOESM1_ESM.docx]

**Supplementary Information**

**
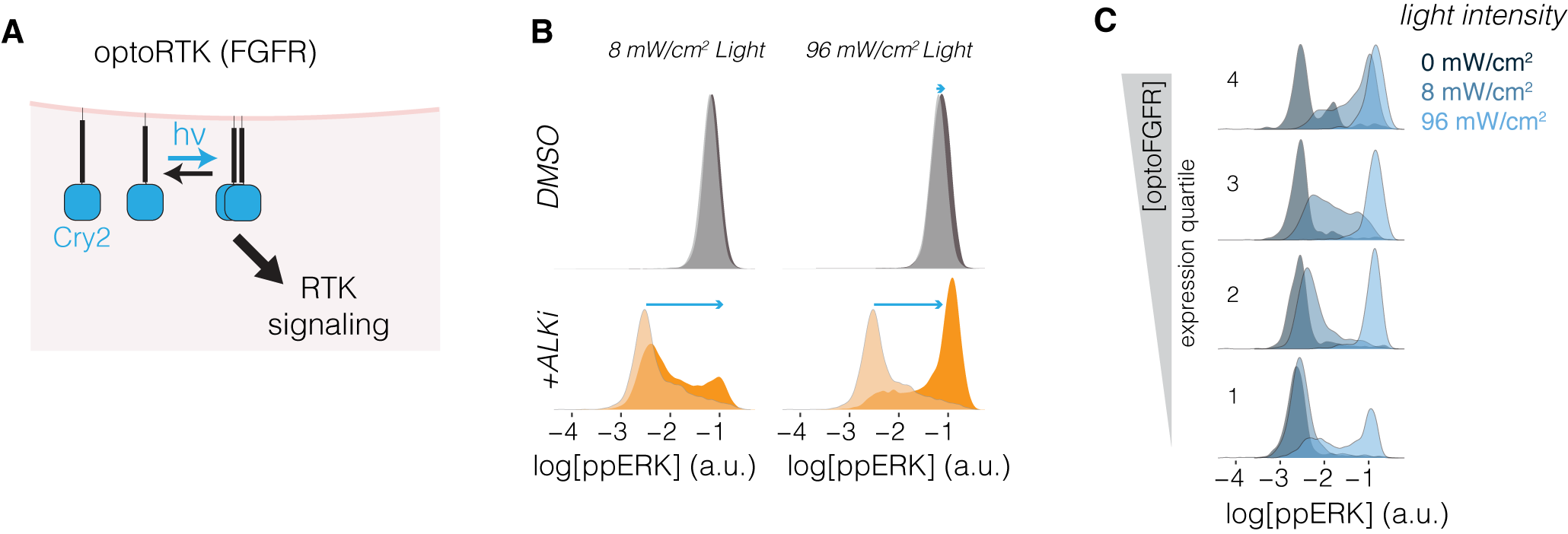
**

**Supplementary Figure 1. OptoFGFR functional profiling of STE-1 cancer cells.** A) optoFGFR comprises the intracellular domain of FGFR1 fused to the PHR domain from Arabidopsis Cryptochrome 2, which clusters under blue light stimulation^75^. The construct is anchored in the membrane through N-terminal myristoylation. B) Single cell distributions of ppERK intensity in STE-1 cells stimulated by the indicated intensity of blue light in the presence (orange) or absence (grey) of ALKi (2 hr pre-incubation of 1µM crizotinib). Strong ppERK response at low light intensity in the presence—but not the absence—of ALKi suggests that ALKi sensitizes cells to RTK stimulation. C) The magnitude of ppERK response in optoFGFR STE-1 cells (here pre-treated with ALKi) is a function of both light intensity and optoFGFR expression levels.


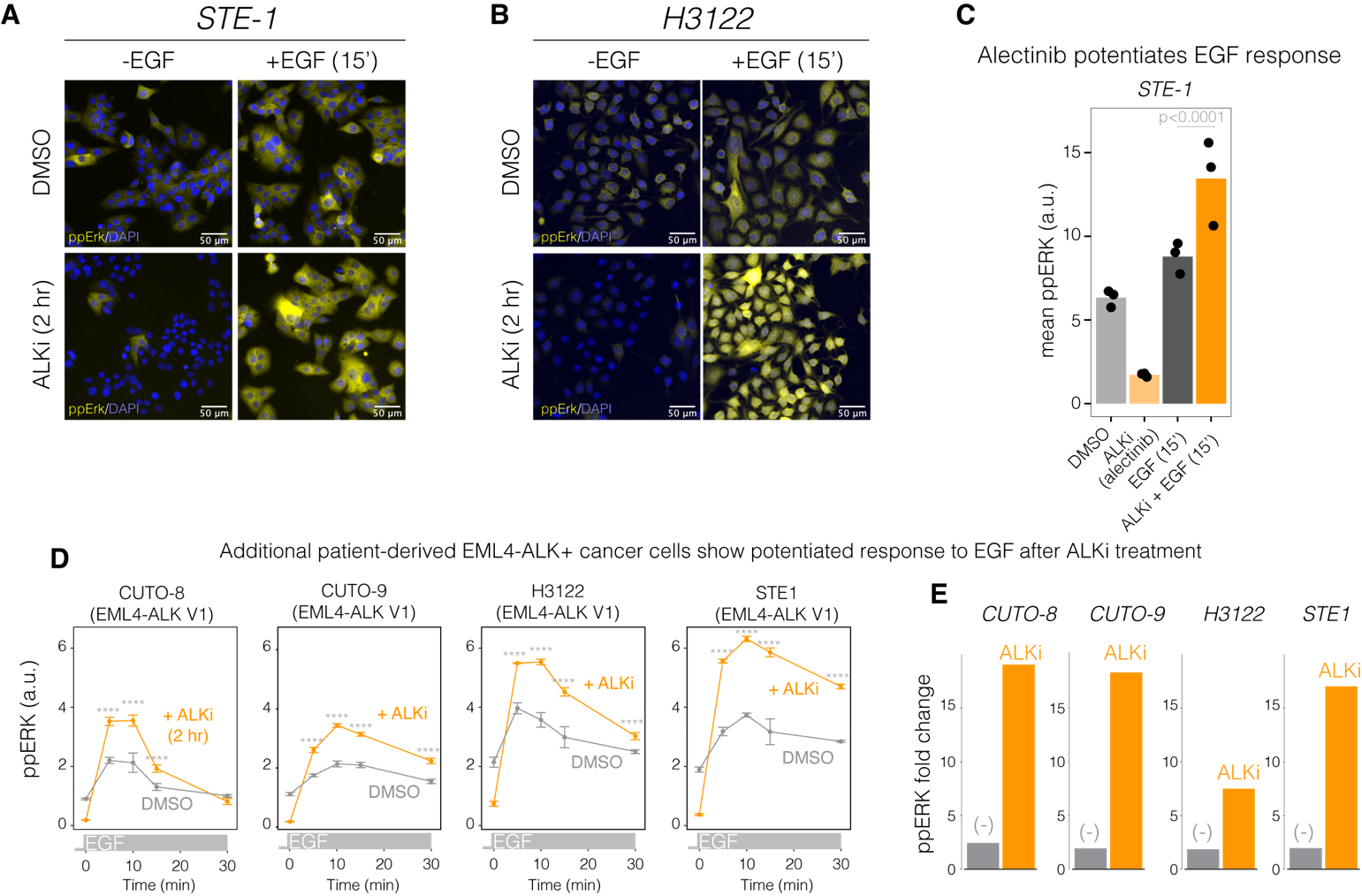


**Supplementary Figure 2. EGF-induced ppERK potentiation in EML4-ALK cancer cell lines.** A,B) Representative images of ppERK staining in STE-1 and H3122 cells in the presence or absence of ALKi (crizotinib, 1 µM) and EGF(50 ng/mL). The +EGF images are reproduced from **Figure 2C.** C) Potentiation of EGFR/ERK signaling in response to EGF (50 ng/mL, 15 min) is also observed using first-line ALKi alectinib (1 µM, 2 hr preincubation). Data points represent means of 1600–5700 STE-1 cells. Significance assessed by one-sided T-test. n = 3 biological replicates. D) Time course of EGF stimulation (50 ng/mL) in two EML4-ALK(V1)+ patient-derived cell lines, CUTO-8 and CUTO-9, compared directly to STE-1 and H3122. ****p<0.0001 comparing ppERK levels in ALKi vs DMSO condition by one-sided Z-test. n = 3 biological replicates. Data points represent mean +/- SEM of 3 biological replicates, with each replicate representing 900-1600 cells for CUTO-8, 200-400 cells for CUTO-9, 4400-6000 cells for H3122, and 3200-4500 cells for STE-1. E) Fold change of ERK response (max ppERK divided by ppERK at T = 0).

**
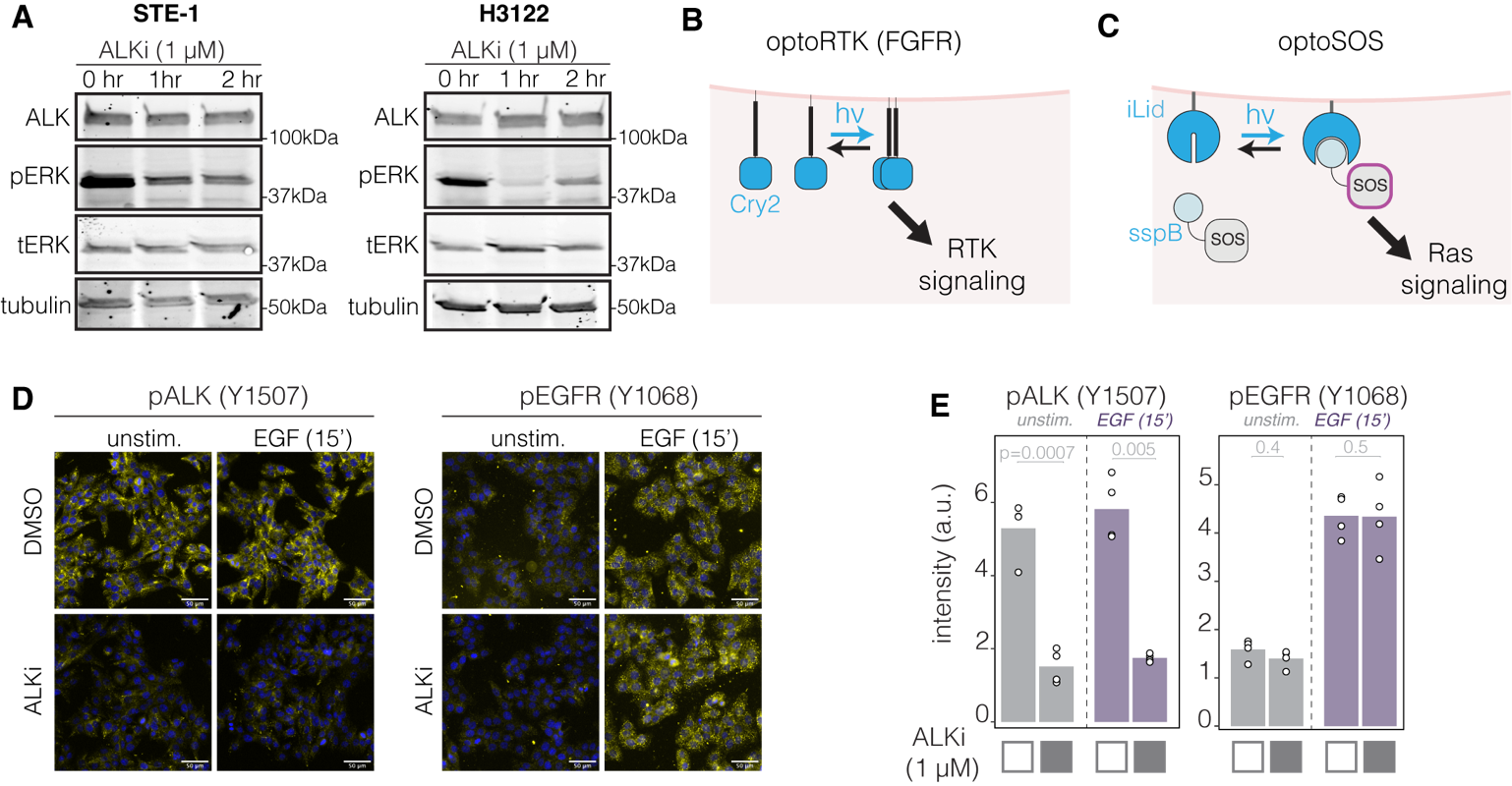
**

**Supplementary Figure 3. Testing hypotheses of RTK/ERK potentiation.** A) Inhibition of EML4-ALK reduces cell MAPK signaling but does not alter total EML4-ALK levels. Cells were cultured in full media with ALKi (crizotinib, 1μM) and processed for SDS-page analysis. B) Comparison of the optoFGFR and optoSOS tools used in **Figure 3**. optoFGFR^31^ allows optogenetic stimulation of the FGFR receptor using light-induced clustering of the Cryptochrome 2 protein^75^. C) OptoSOS allows RAS activation through membrane recruitment of the SOS2 catalytic domain (SOS2_cat_). Membrane recruitment is achieved through blue light dimerization of sspB to the iLid protein^81^ which is anchored to the membrane through a CAAX motif. D) EGFR signal suppression does not result from decreased EGFR phosphorylation. Representative images of immunofluorescence for phospho-ALK(Y1507) and phospho-EGFR(Y1068) in STE-1 cells. E) Quantitation of experiment depicted in (D). ALKi pretreatment did not alter pEGFR levels in either the presence or absence of EGF (50 ng/mL), whereas pALK decreased. Data points represent mean intensity of 320-5300 STE-1 cells. Significance determined by one-sided Z-test, n = 4 biological replicates.


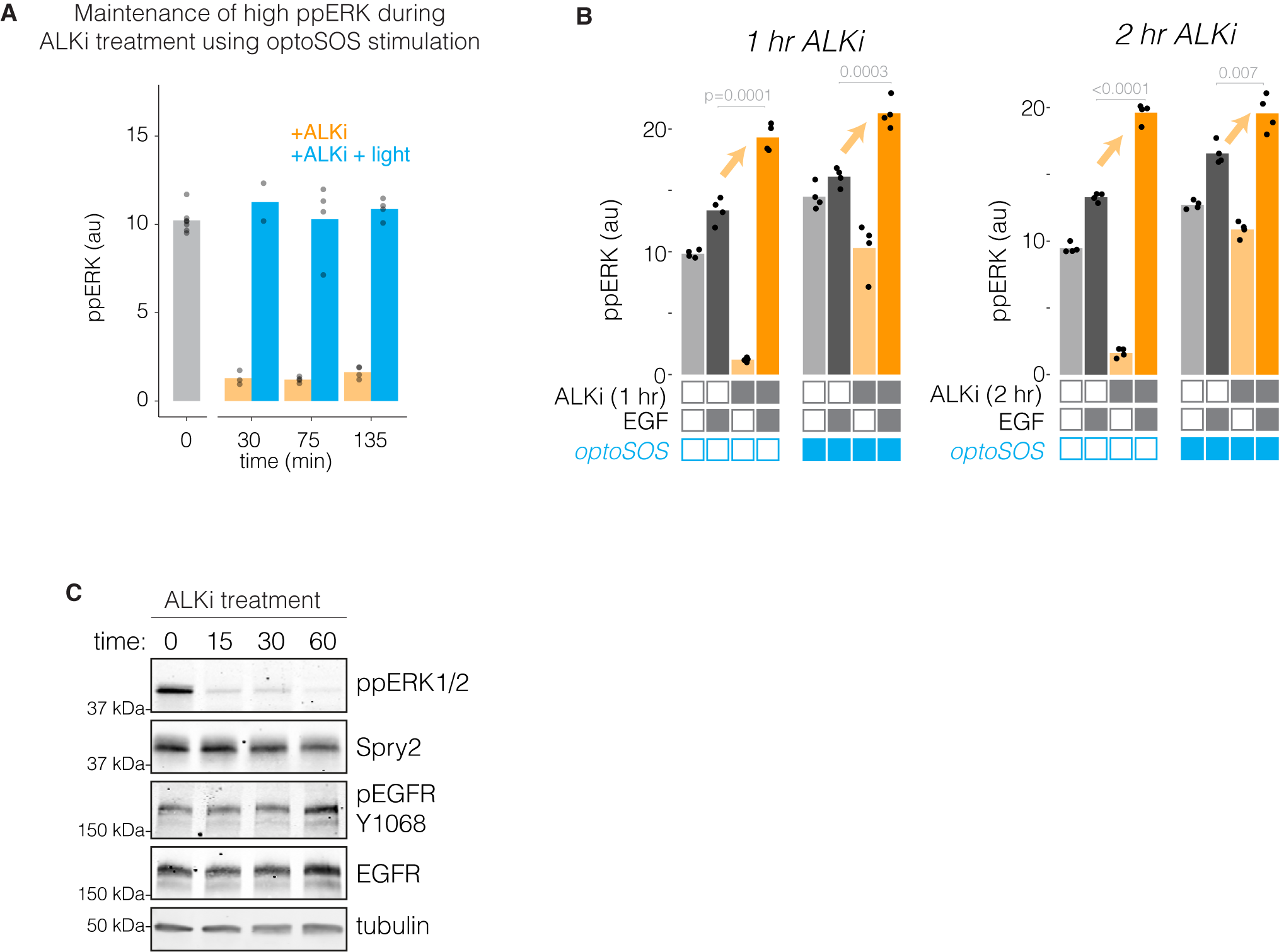


**Supplementary Figure 4**. **Determining the role of ERK-dependent negative feedback on RTK suppression.** A) optoSOS stimulation during ALKi treatment drives ppERK levels comparable to those driven by endogenous EML4-ALK in untreated STE-1 cells. Cells were either untreated (grey) or treated with simultaneous crizotinib (1 µM) and optoSOS (blue light) for the indicated times, and subsequently analyzed for ppERK levels through immunofluorescence. Data is same as (-EGF) data from **Figure 3J and S4B.** B) ppERK response in STE-1 cells treated with ALKi or ALKi + optoSOS for 1 hr or 2 hrs before EGF stimulation (15 min, 50 ng/mL), as in **Figure 3J** but with longer ALKi/light pretreatment. Data in (A) and (B) represent signal from top 25% of optoSOS-expressing cells. Data points represent mean of 154-685 cells per replicate. Significance assessed by one-sided T-test, n = 3 biological replicates. C) Western blot showing levels of negative regulator Spry2 over the first hour of ALKi treatment (crizotinib, 1μM). Despite loss of ERK activity, Spry2 levels remain unchanged over 1hr of ALKi treatment.

**
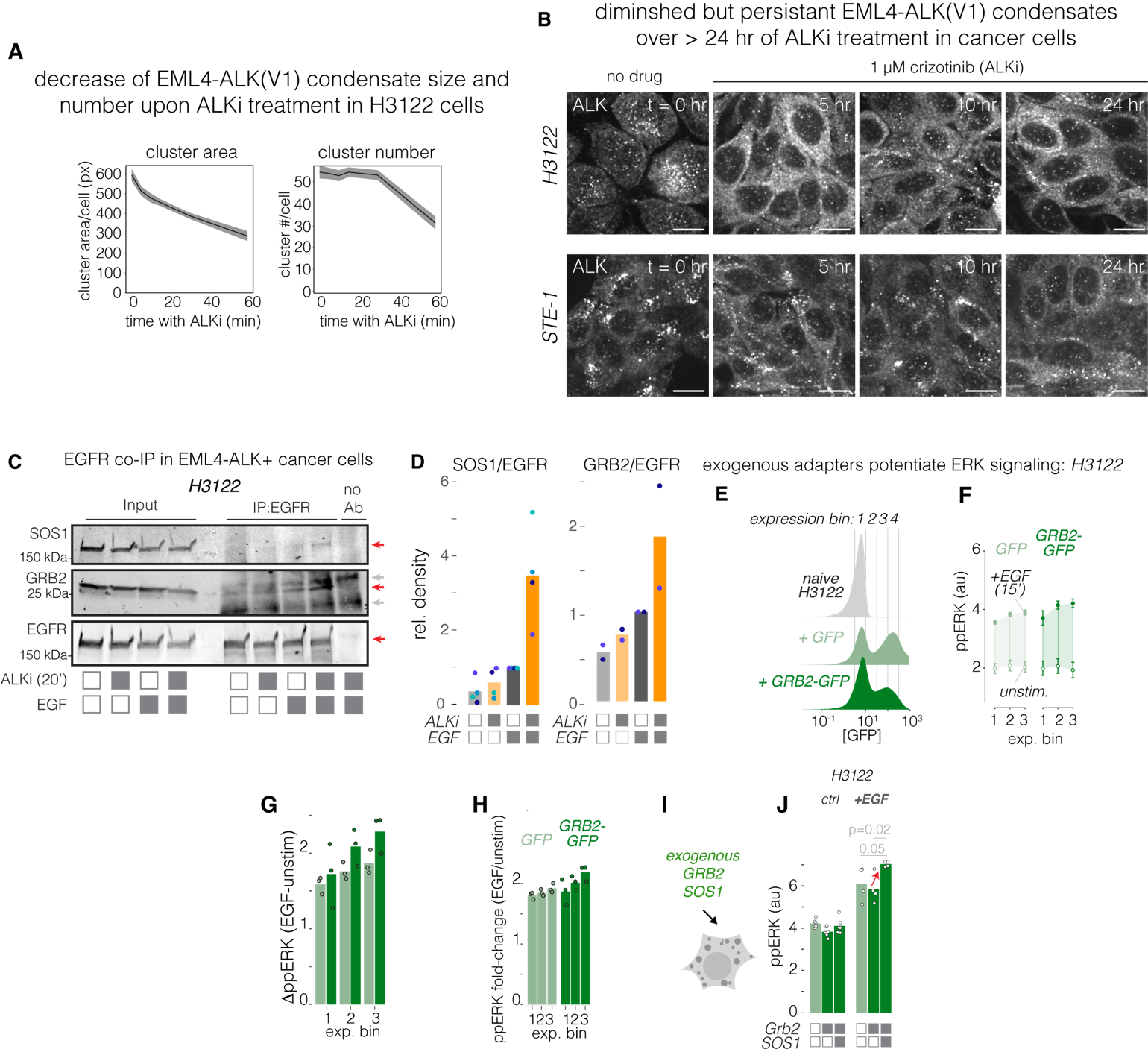
**

**Supplementary Figure 5. Persistence of EML4-ALK(V1) condensates and relief of suppression through GRB2 supplementation.** A) Quantification of ALK condensate size upon treatment with crizotinib (1 µM). Both ALK condensate area and size diminish, but do not disappear, within 1 hr of drug treatment. Data represent mean, ribbons = 95% CI of 1200-1800 cells per data point. B) Representative images of ALK immunofluorescence showing that EML4-ALK condensates persist even after 24 hrs of ALKi treatment, though the number and size of condensates is diminished. Scale bar = 20 µm. C) Immunoprecipitation of EGFR shows enhanced co-precipitation of GRB2 and SOS1 in the presence of both ALKi pretreatment and EGF in H3122 cells. Red arrows: specific bands; grey arrows: non-specific bands. D) Densitometry quantification of independent pulldown experiments. Data points represent independent relative density measurements (SOS1 or GRB2 relative to EGFR), colors represent independent experiments. E) Expression levels of GRB2-GFP or GFP analyzed in (F-H). F) ppERK levels in the absence (open circles) or presence (closed circles) of EGF stimulation (50 ng/mL) as a function of expression levels of the indicated transgene. Data represent mean ppERK intensity +/- SEM of 4 biological replicates, each representing the mean of 100-300 cells. G) Absolute magnitude of ppERK increase for each expression bin from data shown in (F). H) Fold-change of response calculated from data in (G). I) Testing effects of co-expression of GRB2 and SOS1 in H3122 cells. J) H3122 cells transiently co-transfected with GRB2-GFP and SOS1 show higher ppERK in response to EGF compared to the same cells co-transfected with GRB2-GFP and a control plasmid, or with GFP and a control plasmid (see **Methods** for full expression strategy). Data points represent mean of 100-400 cells. Significance determined by one-sided T test, n = 4 biological replicates.


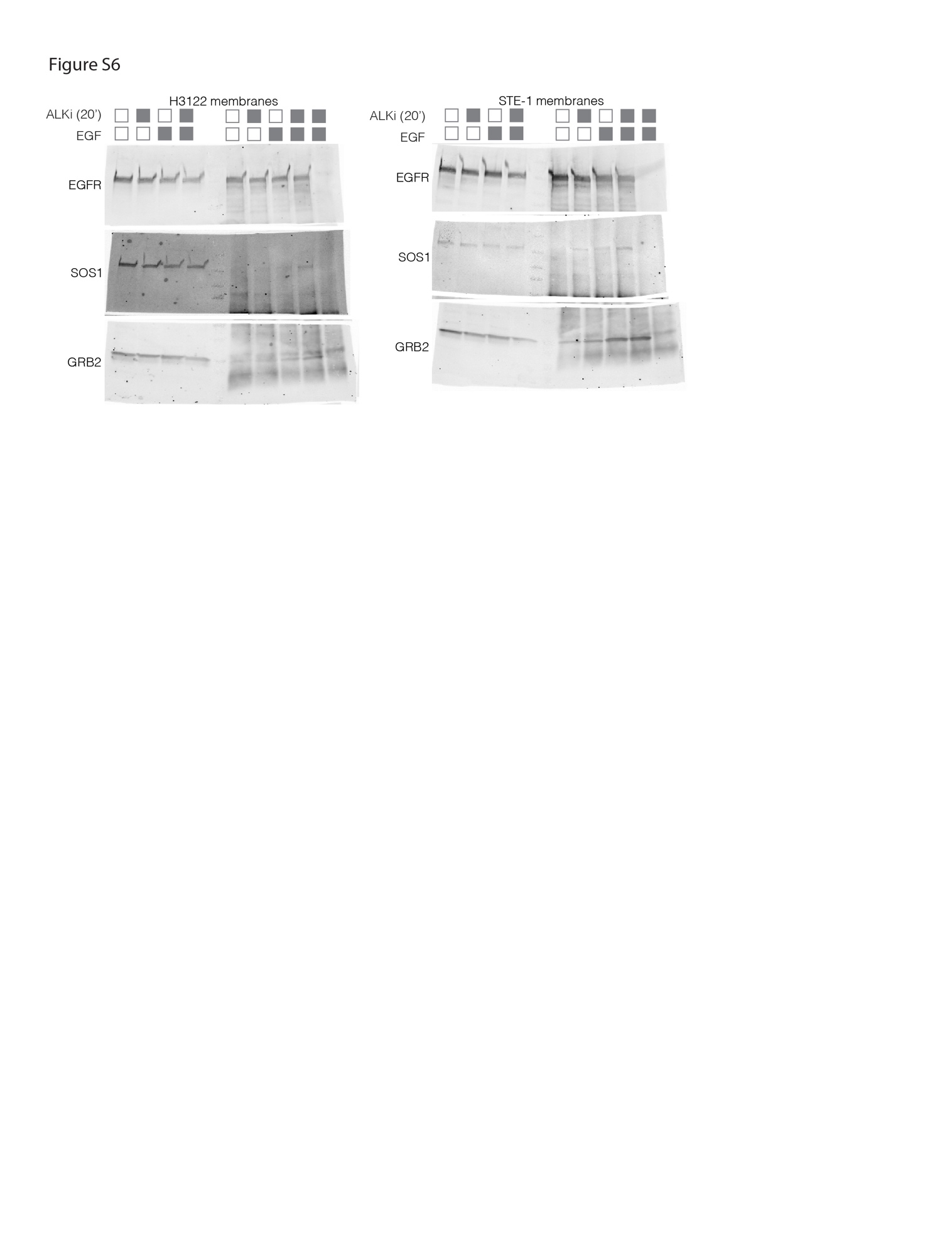


**Supplementary Figure 6. Co-immunoprecipitation of EGFR with GRB2 and SOS1.** Uncropped membranes for H3122 or STE-1 cells starved overnight, pretreated with crizotinib (1 µM for 20 minutes), and stimulated with EGF for 2 minutes before lysis and immunoprecipitation. Cropped membranes are shown in **Figure 4J** and **Figure S5C**.


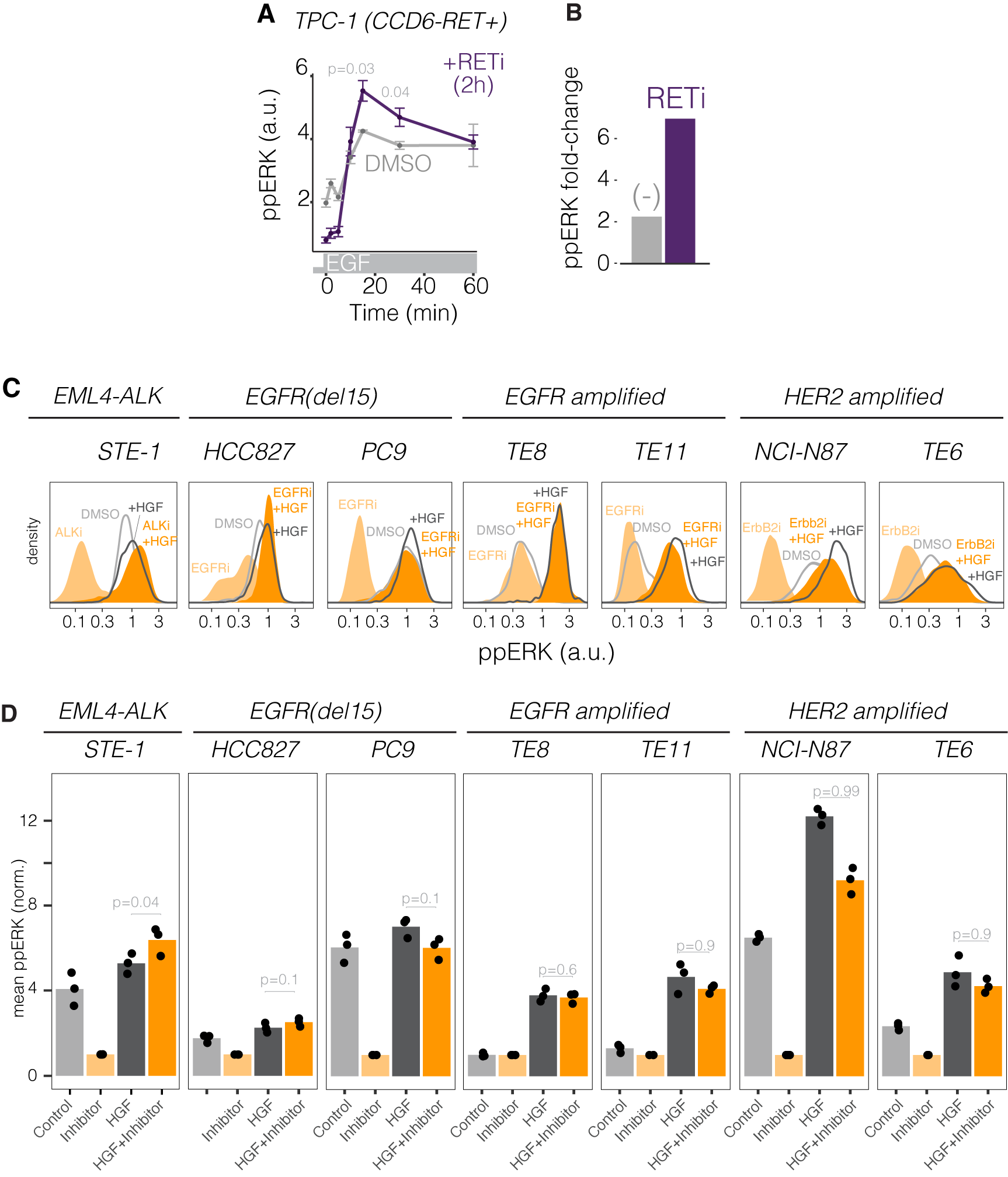


**Supplementary Figure 7. RTK/ERK signal suppression in cancer cells driven by CCDC6-RET but not by hyperactive transmembrane RTK mutants.** A) TPC-1 cancer cells harbor CCDC6-RET, a distinct RTK fusion that forms condensates. Plot shows ppERK response to EGF stimulation after 2 hr pre-treatment with DMSO (grey) or 100 nM BLU-667 (purple), a RET inhibitor. Data represent mean ppERK intensity ± SEM of 3 biological replicates, each representing 660-1400 cells. Significance assessed by one-sided T-test. B) Quantification of ppERK fold-change from (A) shows that oncogene inhibition enhances signaling through EGFR. C,D) To test if oncogenic transmembrane RTKs could suppress non-mutated transmembrane RTK signals in the same cell, we tested ERK response to 15 minute stimulation of hepatocyte growth factor (HGF) in a panel of cancer cell lines harboring either an EGFR point mutation or EGFR or HER2 amplification, in the presence or absence of inhibitors of ALK (alectinib, 1 µM, for STE-1), EGFR (erlotinib, 2 µM, for HCC827, PC9,TE8, TE11) or HER2 (lapatinib, 10 µM), for NCI-N87, TE6). ERK suppression was observed in EML4-ALK cells but not in the cells with oncogenic transmembrane RTKs, consistent with a requirement for cytoplasmic condensates. Panels in (C) show representative single-cell data from each condition. Panels in (D) show means of triplicates, each representing of 1780-5839 STE1 cells, 1174-2328 HCC827 cells, 593-1086 PC9 cells, 286-533 TE8 cells, 678-1655 TE11 cells, 1122-4897 NCI-N87 cells, and 1151-3013 TE6 cells. Significance determined by one-sided T-test, n = 3 biological replicates.


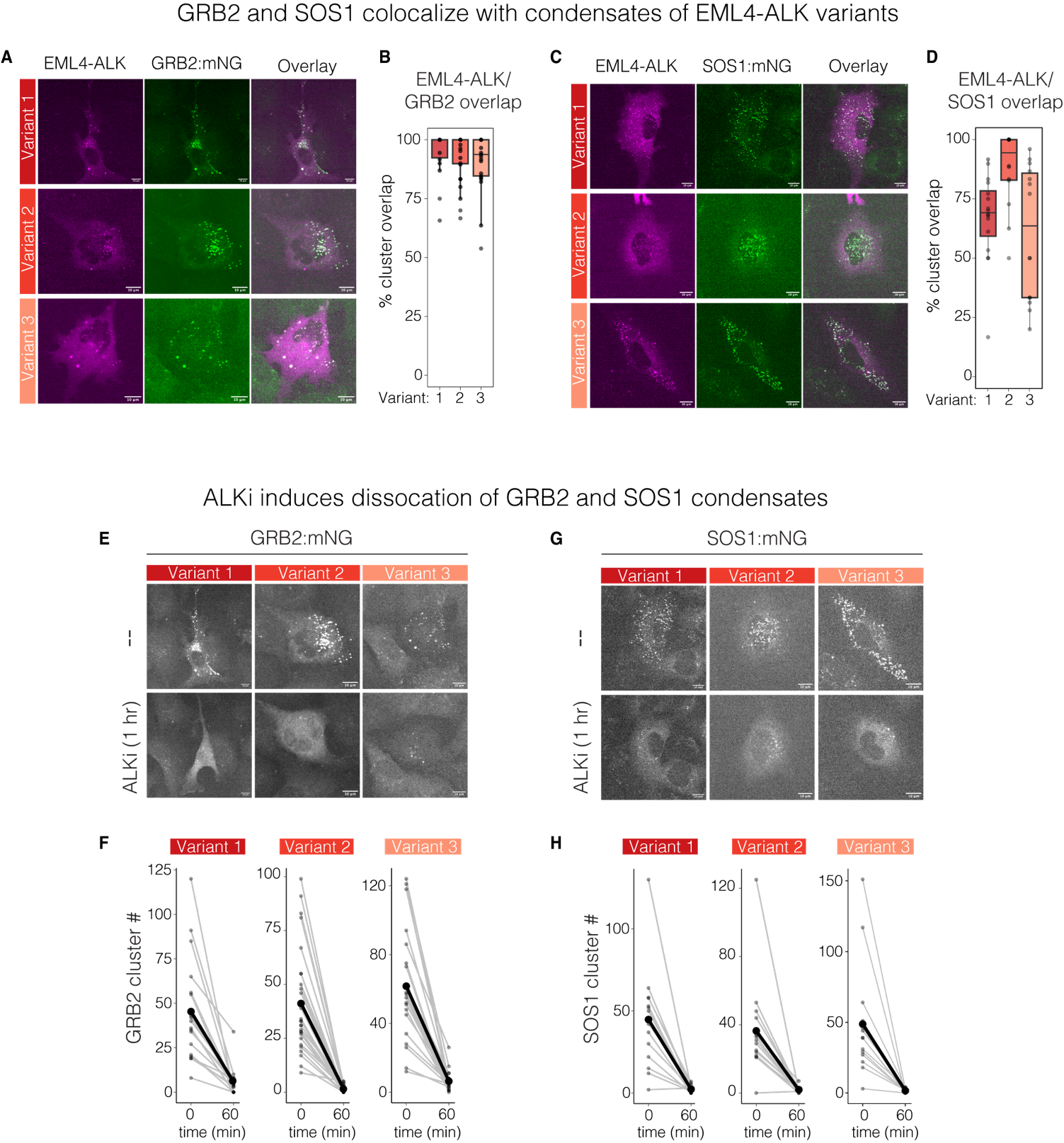


**Supplementary Figure 8. Crizotinib treatment solubilizes endogenous GRB2 and SOS1 condensates in Beas2B cells expressing exogenous EML4-ALK variants.** A) Expression of mCh-EML4-ALK variants in GRB2:mNG Beas2B cells. B) Quantification of the percent mCh-EML4-ALK clusters that overlap with GRB2:mNG clusters. C) Expression of mCh-EML4-ALK variants in SOS1:mNG Beas2B cells. D) Quantification of the percent mCh-EML4-ALK puncta that overlap with SOS1:mNG puncta. Data points represent single cells. Boxplots in (B,D) indicate median and upper/lower quartile, and whiskers show 1.5*IQR. E) ALKi treatment (crizotinib, 1 µM) induces loss of endogenous GRB2 puncta for all variants. F) Quantification of data represented in (E). G) ALKi treatment (crizotinib, 1 µM) induces loss of endogenous SOS1 puncta for all variants. H) Quantification of data represented in (G). Grey lines in (F,G) represent responses of single cells, black line represents mean.

**
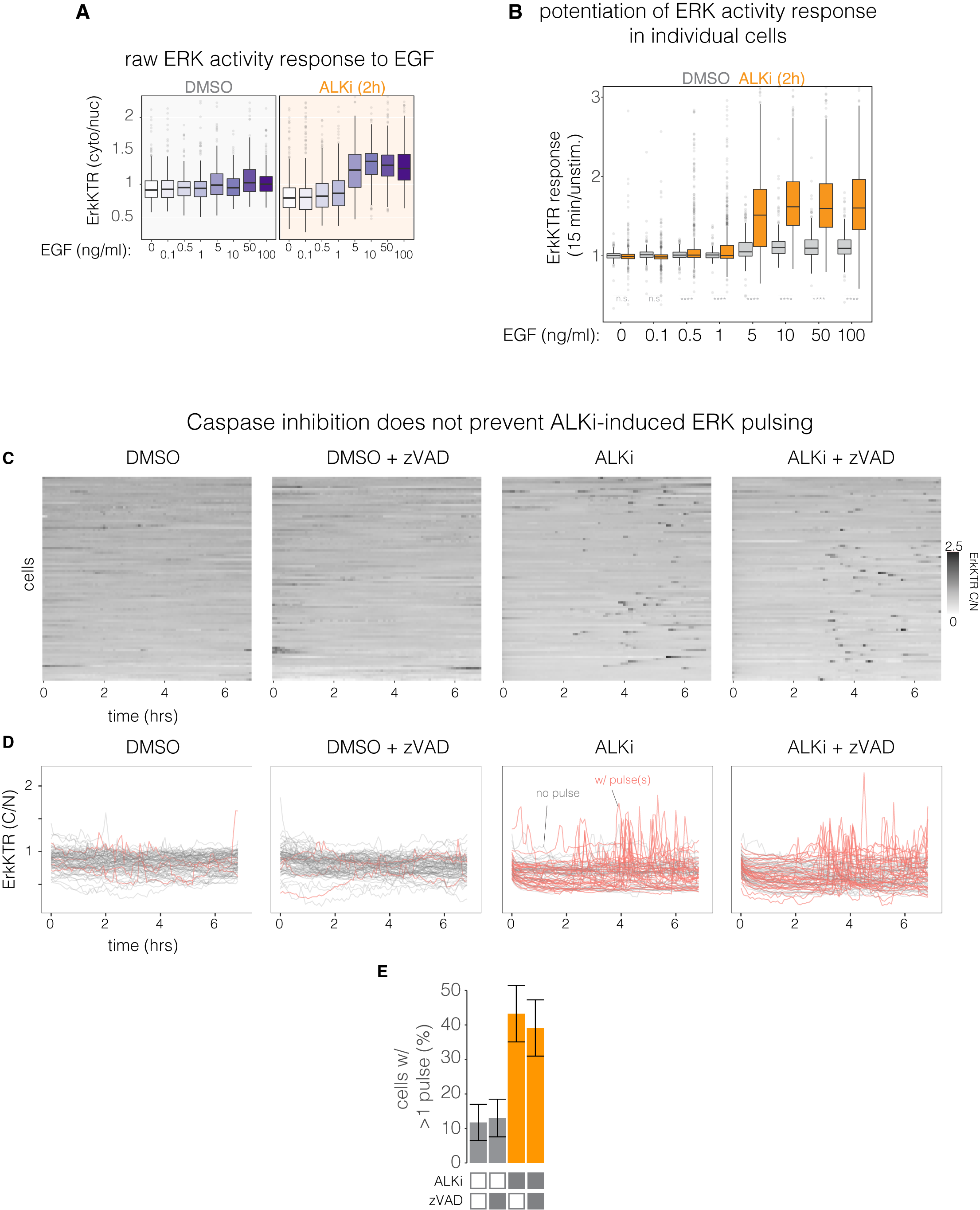
**

**Supplementary Figure 9. ALKi sensitizes ERK activity in STE-1 cancer cells.** A) Single-cell distributions of ERK activity (ErkKTR cytoplasmic/nuclear ratio) in STE-1 cells treated with the indicated concentrations of EGF for 15 minutes, in the presence or absence of ALKi (1 µM crizotinib). Time-lapse videos shown in **Supplementary Movie 5.** B) Single-cell fold-change responses (internally normalized to their value at T = 0). Data is same as in **Figure 6B** with different comparisons. At EGF concentrations with measurable increase in ERK activity (EGF > 0.5 ng/mL), fold-changes of response are larger in cells that were pretreated with ALKi . Boxplots indicate median and upper/lower quartile, and whiskers show 1.5*IQR. ****p<0.0001 by one-sided T-test, n = 316, 473, 384, 727, 421, 536, 285, 595, 307, 541, 256, 425, 294, 439, 421, 415 cells (by order of appearance on B). C,D) Testing the necessity of apoptosis for ERK pulses. Plots show ERK activity upon addition of ALKi (1 µM crizotinib) to cells pretreated for 2 hours with the pan-caspase inhibitor Z-VAD-FMK (50 nM), which inhibits the final stages of apoptosis. Despite preventing cell death (See **Supplementary Movie 5**), caspase inhibition did not suppress ERK pulses, consistent with previous findings that apoptosis-associated EGF ligand secretion occurs downstream of mitochondrial outer membrane permeabilization but upstream of caspase activation. E) Quantification of ERK activity pulses shows no difference in either the presence or absence of caspase inhibition. Data percentage of cell population and 95% CI.

**
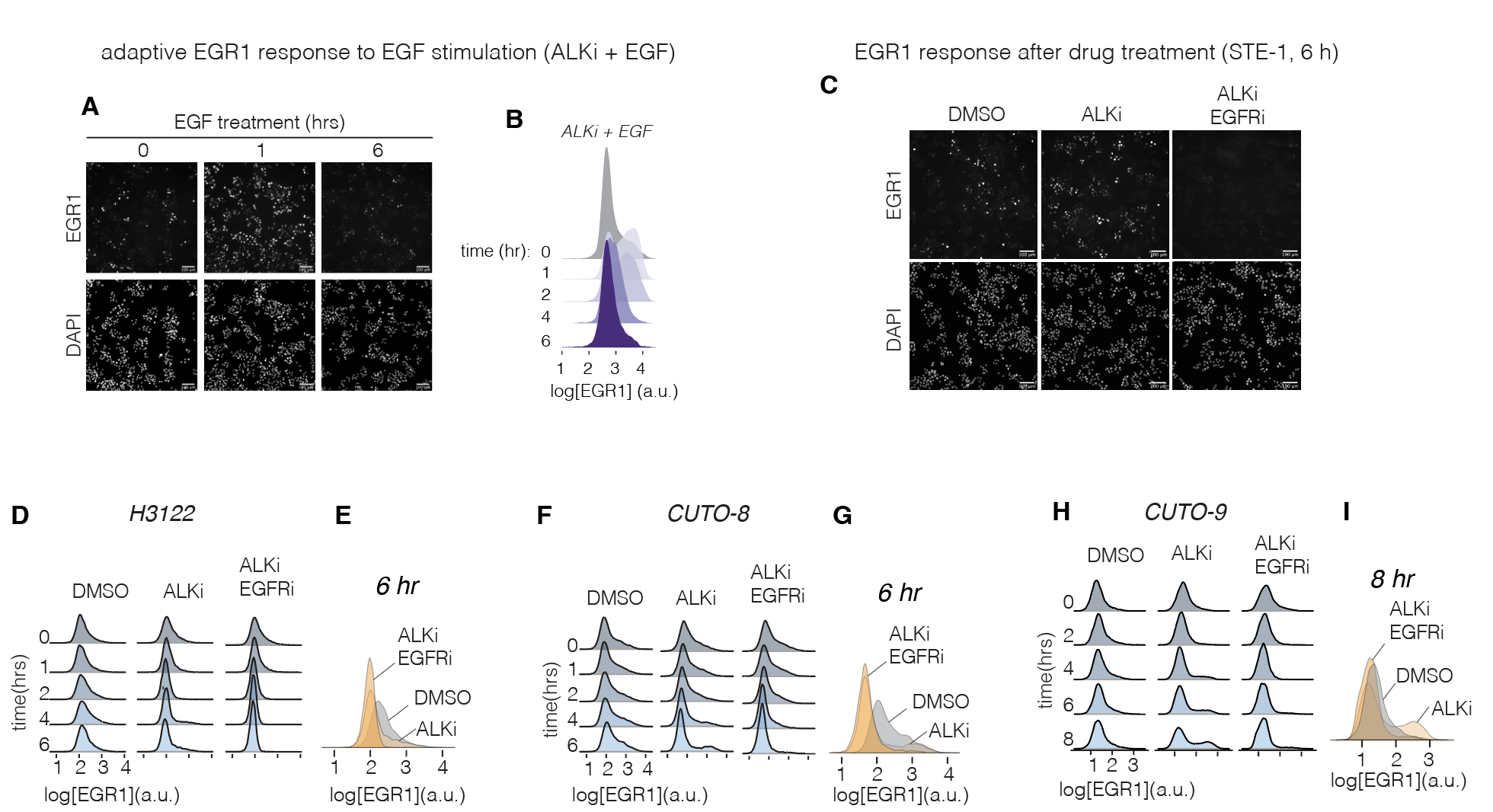
**

**Supplementary Figure 10. Measuring EGR1 expression resulting from ALKi-induced RTK sensitization and paracrine signaling.** A) Representative imaging and single-cell quantitation of EGR1 levels in response to treatment with EGF (50 ng/mL) + ALKi (1 µM crizotinib) in STE-1 cells. B) Quantitation of EGR1 expression from experiment depicted in (A). C) Representative images of EGR1 expression resulting after 6 hr of treatment with the indicated drugs, in the absence of exogenous stimulation. See **Figure 7B,C** for quantitation. D-I) Time course experiments examining EGR1 expression after drug treatment (ALKi: 1 µM crizotinib; EGFRi: 1 µM erlotinib) in H3122 (D,E), CUTO-8 (F,G), and CUTO-9 (H,I) cell lines. All cell lines show an acute increase in EGR1 expression after ALKi treatment, but not after ALKi/EGFRi co-treatment, consistent with activation of paracrine signaling after ALKi monotherapy in EML4-ALK+ cancer cells, as seen in STE-1 cells (**Figure 7A-C).**

**
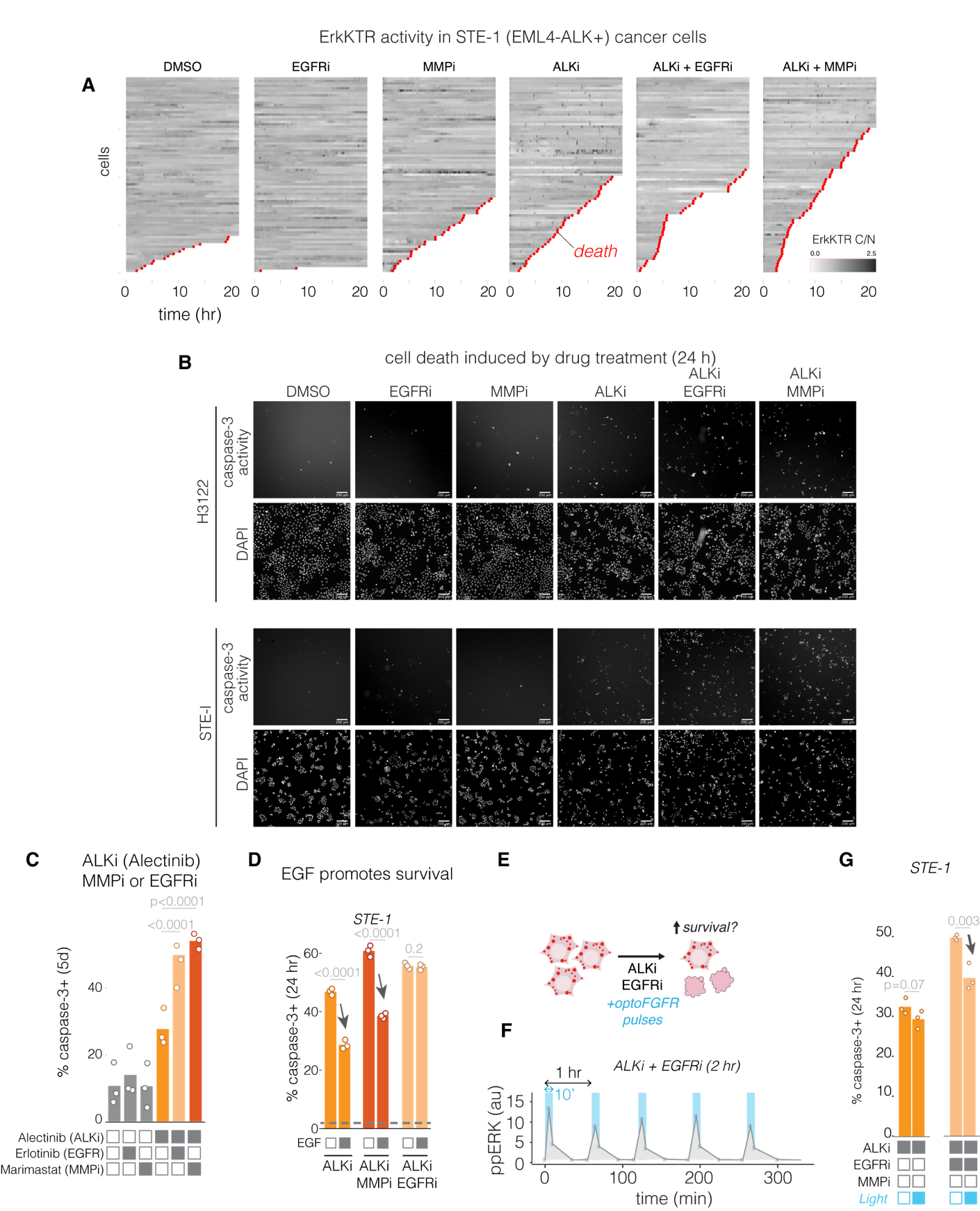
**

**Supplementary Figure 11. Rapid reactivation of EGFR/ERK signaling promotes cancer cell survival.** A) Visualization of ERK activity pulses (ErkKTR) and cell fate over the first 22 hr of treatment with the indicated drug combinations in STE-1 cells. Pulse frequency in living vs dying cells is quantified in **Figure 7E.** Due to bias in selection of individual cells to analyze, the relative proportions of cell death are not indicative of the true death rate. For true death rate, see caspase-3 activation in **Figure 7F**. B) Representative images of caspase-3 activity (Nucview) as quantified in **Figure 7F.** C) Alectinib enhances cell death after 5 days of treatment in STE-1 cells. Each data point represents % caspase3+ cells from 2000-7000 cells. D) EGF (50 ng/mL) promotes survival during ALKi treatment (1 µM crizotinib) and counteracts enhanced killing from ALKi/MMPi (10 µM marimastat) co-treatment, but not from ALKi/EGFRi (1 µM erlotinib) co-treatment. Each data point represents % of caspase-3+ cells from 2000-3000 cells. Dotted line is cell killing from DMSO control treatment. Significance determined using one-sided T-test, n = 3 biological replicates. See **Methods** for more details. E) We tested whether optoFGFR stimulation could reduce the enhanced cell death response in STE-1 cells co-treated with ALKi and EGFRi. F) A 10 min pulse of blue light (96 mW/cm^2^) every hour produced strong, periodic ppERK levels. Data points represent mean of 2 biological replicates, each representing 1260-2130 cells. G) Hourly pulses of optoFGFR/ERK (10 min of 96 mW/cm^2^) reduced cell death in both ALKi and ALKi/EGFRi treated cells, with stronger magnitude of reduction under the co-treatment. Each data point represents 4500-5700 cells. Significance determined using one-sided T-test, n = 3 biological replicates.

**
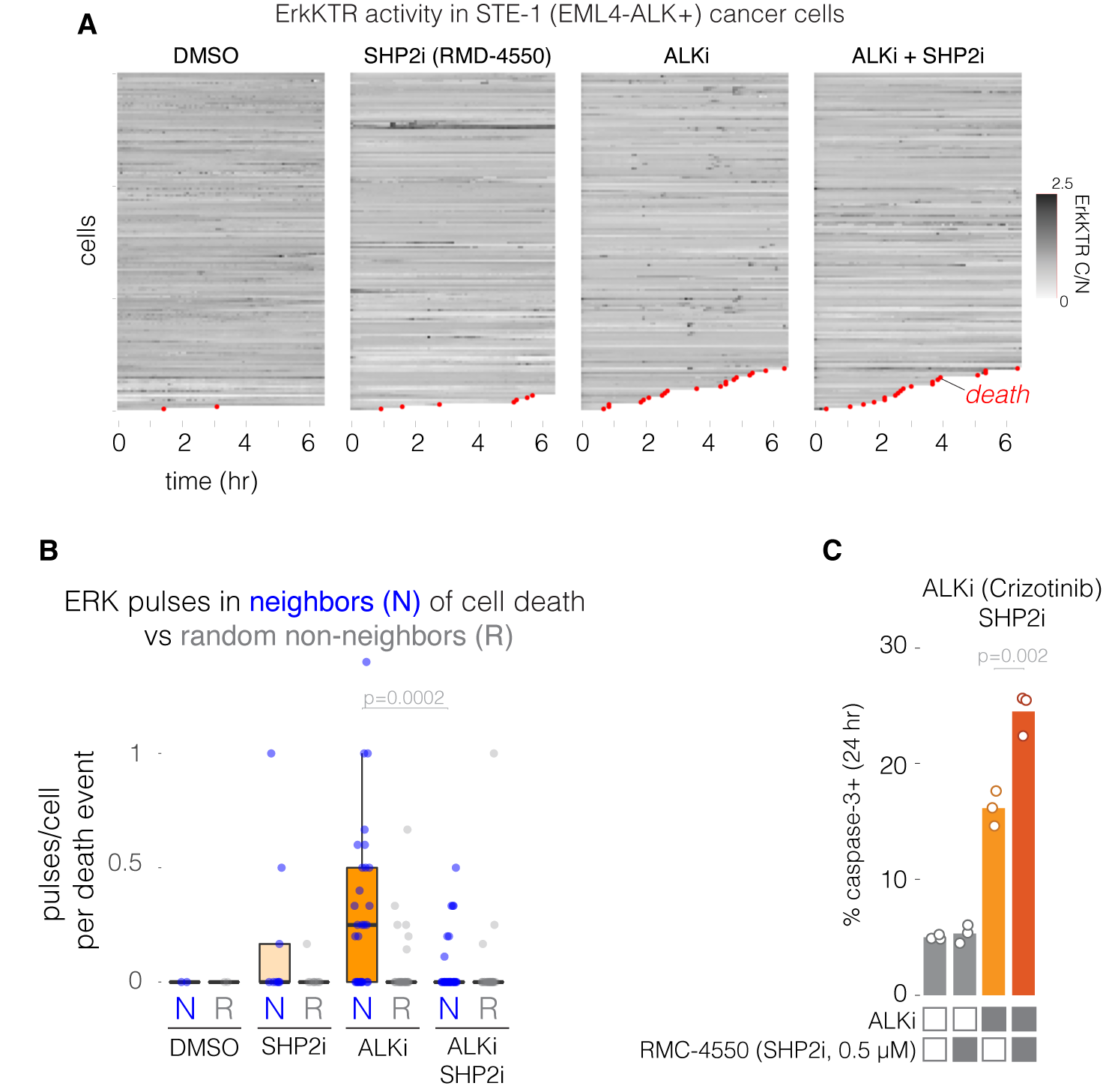
**

**Supplementary Figure 12. The ALKi/SHP2i combination suppresses RTK/ERK reactivation and potentiates acute killing**. A) Visualization of ERK activity pulses (ErkKTR) and cell fate over the first 6 hr of treatment with ALKi (1µM crizotinib), SHP2i (RMD-4550, 1 µM), or their combination, in STE-1 cells. B) Quantitation of ERK pulses in neighbors of dying cells, calculated as depicted in **Figure 6J.** SHP2i suppresses ALKi-induced ERK pulses near dying cells. Boxplots indicate median and upper/lower quartile, and whiskers show 1.5*IQR. Significance assessed by one-sided T-test. C) ALKi (1 µM crizotinib) and SHP2i combination treatment potentiate cell death within 24 hrs of treatment in H3122 cells. Data points each represent 22,000-25,000 cells. Significance assessed by one-sided T-test, n = 3 biological replicates.

**Supplementary Table 1. Biological and experimental replicate number for each experiment.**

| **Figure** | **biological replicates** | **experimental replicates** |
| --- | --- | --- |
| 1B | 3 | 2 |
| 1F | single cell analysis | 4 |
| 1G | 2 | 4 |
| 2A | 3 | 3 |
| 2E-F |  | |
| STE-1 | 3 | 3 |
| H3122 | 3 | 3 |
| CUTO-8 | 3 | 2 |
| CUTO-9 | 3 | 2 |
| Kelly | 3 | 3 |
| Sy5y | 3 | 3 |
| 2H,I | 3 | 3 |
| 3B | 3 | 3 |
| 3E | 4 | 3 |
| 3J | 4 | 2 |
| 4A | 3 | 3 |
| 4B | single cell analysis | 2 |
| 4 D-I | single cell analysis | 3 |
| 4 J,K | whole cell lysate | 3 |
| 4 M-P | 3 | 4 |
| 5B-J | single cell analysis | 2 |
| 5L,M | 3 | 4 |
| 6 B | single cell analysis | 3 |
| 6 C-L | single cell analysis | 3 |
| 7 B,C | single cell analysis | 2 |
| 7 E | single cell analysis | 3 |
| 7 F | 3 | 3 |
| 7 G,H | 4 | 2 |
| S1 B,C | single cell analysis | 4 |
| S2 C | 3 | 2 |
| S2 D,E |  | |
| CUTO-8 | 3 | 2 |
| CUTO-9 | 3 | 2 |
| H3122 | 3 | 3 |
| STEI | 3 | 3 |
| S3 A | Whole cell lysate | 3 |
| S3 D,E | 4 | 2 |
| S4 A,B | 4 | 2 |
| S4 C | whole cell lysate | 3 |
| S5 A,B | single cell | 3 |
| S5 C,D | whole cell lysate | 2,4 |
| S5 E-H | 3 | 3 |
| S5 J | 4 | 3 |
| S6 | single cell analysis | 3 |
| S7 A,B | 3 | 2 |
| S7 C-D | 3 | 3 |
| S8 | single cell analysis | 3 |
| S9 A,B | single cell analysis | 2 |
| S9 C-E | single cell analysis | 3 |
| S10 | single cell analysis | 2 |
| S11 A | single cell analysis | 3 |
| S11 B | 3 | 3 |
| S11 C | 3 | 2 |
| S11D | 3 | 3 |
| S11 F-G | 3 | 3 |
| S12 A,B | single cell analysis | 3 |
| S12 C | 3 | 1 |
