# Supplementary material for: Oncogenic EML4-ALK assemblies suppress growth factor perception and modulate drug tolerance
Source: Nat Commun. 2024 Nov 2;15:9473. doi: 10.1038/s41467-024-53451-7 (PMC11531495; doi:10.1038/s41467-024-53451-7)
Supplement: Supplementary file 7 — Description of Additional Supplementary Files [file 41467_2024_53451_MOESM7_ESM.pdf]

Description of Additional Supplementary Material Files

Supplementary Movie 1. Suppression of GRB2 membrane translocation in the presence of EML4-ALK(V1) GRB2-mNG2 localization in response to EGF (50 ng/mL) stimulation in Beas2B cells where GRB2 is tagged at the endogenous locus (*GRB2:mNG2*). Cells were transiently transfected with EML4-ALK(V1) such that only some cells expressed the construct. Arrows denote cells that do or do not express EML4-ALK(V1).

Supplementary Movie 2. GRB2 dissociates from condensates upon ALK inhibition. inhibition. GRB2-mNG2 localization in response to ALK inhibitor crizotinib (1 μM). Arrows indicate cells that express EML4-ALK (V1).

Supplementary Movie 3. EML4-ALK inhibition sensitizes STE1 cells to lower concentrations of EGF stimulation. ErkKTR response in STE-1 cells in responst to EGF stimulation at concentrations ranging from 0.5-50 ng/mL following 2 hrs of DMSO (top row) or ALKi (bottom row) pretreatment. Video is representative of quantification presented in Figure. 6B.

Supplementary Movie 4. Spontaneous ErkKTR pulsing after ALK inhibition depends on EGFR and MMPs. ErkKTR activity in STE-1 cells in response to DMSO, ALKi (1 μM crizotinib) or ALKi in combination with EGFRi (1 μM erlotinib) or ALKi+MMPi (10 μM marimastat). Videos are representative of quantification presented in Figure 6C-L. Time is in hh:mm.

Supplementary Movie 5. Caspase inhibition does not decrease ALKi-induced Erk activity pulses. ErkKTR activity (top row) and respective H2B-iRFP fluorescent nuclei (bottom row) in STE-1 cells in response to treatment with ALKi (crizotinib,1 μM) or ALKi in combination with pan-caspase inhibitor (z-VAD 50 nM). Pan-caspase inhibitor was added 2 hours prior to onset of experiment, and ALKi was added with onset of imaging. Video is representative of quantification Figure S8 C-E.
