# Supplementary material for: Oncogenic EML4-ALK assemblies suppress growth factor perception and modulate drug tolerance
Source: Nat Commun. 2024 Nov 2;15:9473. doi: 10.1038/s41467-024-53451-7 (PMC11531495; doi:10.1038/s41467-024-53451-7)
Supplement: Supplementary file 9 — Reporting Summary [file 41467_2024_53451_MOESM9_ESM.pdf]

nature portfolio

Corresponding author(s): *Double-blind peer review submissions: write DBPR and your manuscript number here instead of author names.*

Last updated by author(s): Jul 4, 2024

Reporting Summary

Nature Portfolio wishes to improve the reproducibility of the work that we publish. This form provides structure for consistency and transparency in reporting. For further information on Nature Portfolio policies, see our Editorial Policies and the Editorial Policy Checklist.

Statistics

For all statistical analyses, confirm that the following items are present in the figure legend, table legend, main text, or Methods section.

n/a	Confirmed	
☐	☒	The exact sample size (n) for each experimental group/condition, given as a discrete number and unit of measurement
☐	☒	A statement on whether measurements were taken from distinct samples or whether the same sample was measured repeatedly
☐	☒	The statistical test(s) used AND whether they are one- or two-sided<br>*Only common tests should be described solely by name; describe more complex techniques in the Methods section.*
☒	☐	A description of all covariates tested
☐	☒	A description of any assumptions or corrections, such as tests of normality and adjustment for multiple comparisons
☐	☒	A full description of the statistical parameters including central tendency (e.g. means) or other basic estimates (e.g. regression coefficient) AND variation (e.g. standard deviation) or associated estimates of uncertainty (e.g. confidence intervals)
☐	☒	For null hypothesis testing, the test statistic (e.g. F, t, r) with confidence intervals, effect sizes, degrees of freedom and P value noted<br>*Give P values as exact values whenever suitable.*
☒	☐	For Bayesian analysis, information on the choice of priors and Markov chain Monte Carlo settings
☒	☐	For hierarchical and complex designs, identification of the appropriate level for tests and full reporting of outcomes
☒	☐	Estimates of effect sizes (e.g. Cohen's d, Pearson's r), indicating how they were calculated

Our web collection on statistics for biologists contains articles on many of the points above.

Software and code

Policy information about availability of computer code

Data collection	Nikon Elements AR (2019)<br>Arduino IDE (v1.8)<br>BD Influx System
Data analysis	CellProfiler (4.0.7)<br>Ilastik (1.4)<br>MatLab(R2020a)<br>RStudio(1.1.463)<br>dplyr(1.0.7)<br>ggplot2(3.3.5)

For manuscripts utilizing custom algorithms or software that are central to the research but not yet described in published literature, software must be made available to editors and reviewers. We strongly encourage code deposition in a community repository (e.g. GitHub). See the Nature Portfolio guidelines for submitting code & software for further information.

Data

Policy information about availability of data

All manuscripts must include a data availability statement. This statement should provide the following information, where applicable:

- Accession codes, unique identifiers, or web links for publicly available datasets
- A description of any restrictions on data availability
- For clinical datasets or third party data, please ensure that the statement adheres to our policy

Source data are provided with this paper.

Field-specific reporting

Please select the one below that is the best fit for your research. If you are not sure, read the appropriate sections before making your selection.

[x] Life sciences [ ] Behavioural & social sciences [ ] Ecological, evolutionary & environmental sciences

For a reference copy of the document with all sections, see nature.com/documents/nr-reporting-summary-flat.pdf

Life sciences study design

All studies must disclose on these points even when the disclosure is negative.

Sample size	Sample size refers to the number of cells analyzed per well or by number of biological replicates that each datapoint represents. Number of cells were determined using nuclei segmentation. For live cell image quantification of Grb2 aggregates in Beas2B cells, each trace represents a single cell analyzed as described.
Data exclusions	No data points were excluded from analysis
Replication	Experiments were run with 3-4 replicates and/or were performed 3-4 times. All replicates are detailed in Supplementary Figure 1
Randomization	All experiments were performed on cell lines with variable treatments. Thus, randomization was not required
Blinding	No blinding was required because data was analyzed through automated image analysis.

Reporting for specific materials, systems and methods

We require information from authors about some types of materials, experimental systems and methods used in many studies. Here, indicate whether each material, system or method listed is relevant to your study. If you are not sure if a list item applies to your research, read the appropriate section before selecting a response.

Materials & experimental systems

n/a	Involved in the study	
[ ]	[x]	Antibodies
[ ]	[x]	Eukaryotic cell lines
[x]	[ ]	Palaeontology and archaeology
[x]	[ ]	Animals and other organisms
[x]	[ ]	Human research participants
[x]	[ ]	Clinical data
[x]	[ ]	Dual use research of concern

Methods

n/a	Involved in the study	
[x]	[ ]	ChIP-seq
[x]	[ ]	Flow cytometry
[x]	[ ]	MRI-based neuroimaging

Antibodies

Antibodies used	EGFR (CST #4267), pALK(CST #14678); EGR1 (CST #4153); SOS1 (CST #5890), SPRY2 (CST #14954), pERK1/2 (CST #4370), tubulin (CST #3873); GRB2 (Thermo Fisher scientific, PA1-10033). ,IRDye® 800CW Goat anti-Rabbit IgG, LI-COR #926-32211, IRDye® 680RD Donkey anti-Mouse IgGLI-COR, #926-68072, IgG (H+L) Cross-Adsorbed Goat anti-Rabbit, DyLight™ 488, Invitrogen #35553; Goat anti-Rabbit IgG (H+L) Cross-Adsorbed Secondary Antibody, DyLight™ 650, Invitrogen #SA510034
Validation	https://www.cellsignal.com/products/primary-antibodies/egf-receptor-d38b1-xp-rabbit-mab/4267<br>https://www.cellsignal.com/products/primary-antibodies/sos1-antibody/5890<br>https://www.cellsignal.com/products/primary-antibodies/spry2-d3g1a-rabbit-mab/14954<br>https://www.cellsignal.com/products/primary-antibodies/phospho-p44-42-mapk-erk1-2-thr202-tyr204-d13-14-4e-xp-rabbit-mab/4370<br>https://www.thermofisher.com/antibody/product/GRB2-Antibody-Polyclonal/PA1-10033

https://www.cellsignal.com/products/primary-antibodies/a-tubulin-dm1a-mouse-mab/3873
https://www.licor.com/documents/rfm2hw40wf33p06f3ndjrcorwi5usbft
https://www.licor.com/documents/1rur5wg8vsznx2ll28hgimwzjsgrlbsf
https://www.thermofisher.com/order/genome-database/generatePdf?productName=Rabbit%20IgG%20(H+L)%20Cross%20Adsorbed&assayType=PRANT&productId=SA5-10034&detailed=true
https://www.thermofisher.com/antibody/product/Goat-anti-Rabbit-IgG-H-L-Cross-Adsorbed-Secondary-Antibody-Polyclonal/35553
https://www.cellsignal.com/products/primary-antibodies/phospho-alk-tyr1507-d6f1v-rabbit-mab/14678
https://www.cellsignal.com/products/primary-antibodies/egr1-15f7-rabbit-mab/4153?utm_strategy=lev&utm_conv=mon&utm_stage=ous&utm_tactic=ppc&utm_region=hq&gclid=CjwKCAjwkJm0BhBxEiwAwT1AXIWMk1zPxLrM2NOiYaXd0hOPhuWMdoqaVLamhzdzYEKC35IKu3C8VBoCU-wQAvD_BwE&gclsrc=aw.ds

Eukaryotic cell lines

Policy information about cell lines

Cell line source(s)	STE-I; H3122; Beas2B (ATCC #CRL-9609); TPC-1; HEK293T (Takara); SH-SY5Y; KELLY CUTO-8; CUTO-9; PC9; TE8; TE11; NCI-N87; TE6; HCC827
Authentication	Cells were not authenticated after purchase
Mycoplasma contamination	All cell lines were tested for mycoplasma every 3-6 months.
Commonly misidentified lines (See ICLAC register)	none
